# Glycerol amendment enhances biosulfidogenesis in acid mine drainage-affected areas: An incubation column experiment

**DOI:** 10.3389/fbioe.2022.978728

**Published:** 2022-08-29

**Authors:** A. M. Ilin, C. M. van der Graaf, I. Yusta, A. Sorrentino, I. Sánchez-Andrea, J. Sánchez-España

**Affiliations:** ^1^ Department of Geology, Faculty of Science and Technology, University of the Basque Country (UPV/EHU), Barrio Sarriena s/n, Leioa, Spain; ^2^ Laboratory of Microbiology, Wageningen University (WUR), Wageningen, Netherlands; ^3^ ALBA Synchrotron Light Source, Cerdanyola del Vallés, Barcelona, Spain; ^4^ Mine Wastes and Environmental Geochemistry Research Group, Department of Geological Resources for the Ecological Transition, (CN IGME-CSIC), Madrid, Spain

**Keywords:** biosulfidogenesis, *Desulfosporosinus*, sulfate-reducing bacteria, metal sulfide neoformation, acid mine drainage (AMD), mine tailings, incubation column, bioremediation

## Abstract

Microbial sulfate (SO_4_^2−^) reduction in Acid Mine Drainage (AMD) environments can ameliorate the acidity and extreme metal concentrations by consumption of protons via the reduction of SO_4_^2−^ to hydrogen sulfide (H_2_S) and the concomitant precipitation of metals as metal sulfides. The activity of sulfate-reducing bacteria can be stimulated by the amendment of suitable organic carbon sources in these generally oligotrophic environments. Here, we used incubation columns (IC) as model systems to investigate the effect of glycerol amendment on the microbial community composition and its effect on the geochemistry of sediment and waters in AMD environments. The ICs were built with natural water and sediments from four distinct AMD-affected sites with different nutrient regimes: the oligotrophic Filón Centro and Guadiana acidic pit lakes, the Tintillo river (Huelva, Spain) and the eutrophic Brunita pit lake (Murcia, Spain). Physicochemical parameters were monitored during 18 months, and the microbial community composition was determined at the end of incubation through 16S rRNA gene amplicon sequencing. SEM-EDX analysis of sediments and suspended particulate matter was performed to investigate the microbially-induced mineral (neo)formation. Glycerol amendment strongly triggered biosulfidogenesis in all ICs, with pH increase and metal sulfide formation, but the effect was much more pronounced in the ICs from oligotrophic systems. Analysis of the microbial community composition at the end of the incubations showed that the SRB *Desulfosporosinus* was among the dominant taxa observed in all sulfidogenic columns, whereas the SRB *Desulfurispora*, *Desulfovibrio* and *Acididesulfobacillus* appeared to be more site-specific. Formation of Fe^3+^ and Al^3+^ (oxy)hydroxysulfates was observed during the initial phase of incubation together with increasing pH while formation of metal sulfides (predominantly, Zn, Fe and Cu sulfides) was observed after 1–5 months of incubation. Chemical analysis of the aqueous phase at the end of incubation showed almost complete removal of dissolved metals (Cu, Zn, Cd) in the amended ICs, while Fe and SO_4_^2−^ increased towards the water-sediment interface, likely as a result of the reductive dissolution of Fe(III) minerals enhanced by Fe-reducing bacteria. The combined geochemical and microbiological analyses further establish the link between biosulfidogenesis and natural attenuation through metal sulfide formation and proton consumption.

## 1 Introduction

Acidic pit lakes (APL) form upon flooding of abandoned open-pit polymetallic sulfide or coal mines which catalyzes the generation of acid mine drainage (AMD) through oxidative dissolution of metal sulfides in pit walls, waste rock piles and tailings deposits (Abinandan et al., 2018). The extreme acidity (pH 2–3.5; Sánchez-España et al., 2008; Sánchez-España et al., 2020a) and high concentration of heavy metal(oid)s (e.g., up to 36.7 g/L Fe, 6.7 g/L Zn, 1.9 g/L Al, 1.3 g/L Cu, 159 mg/L As, 18 mg/L Cd, 18.7 mg/L Co) pose a severe environmental risk (Sánchez-España et al., 2020a; Sánchez-España et al., 2020b).

These systems have potential for microbially mediated attenuation of metal concentrations and acidity, predominantly through biosulfidogenesis by sulfate-reducing bacteria (SRB; Park and Faivre, 2022; Whitworth et al., 2022). The microbial production of sulfide (H_2_S) in the presence of high metal concentration enables the formation of microscopic metal sulfide aggregates, lowering dissolved metal concentrations (Eq. 1). Because sulfate reduction at low pH is proton (H^+^)-consuming, biosulfidogenesis by SRB induces an increase in pH (Eq. 2). Indirectly this further lowers metal concentration through adsorption and/or precipitation processes (Nordstrom and Alpers, 1999; Kaksonen and Puhakka, 2007). In the case of large (up to several hm^3^ of contaminated water), acidic metalliferous water bodies such as APL, triggering *in-situ* biosulfidogenesis can be a low-maintenance, passive bioremediation strategy, using the APL as large-scale natural reactors (Tuttle et al., 1969; Christensen et al., 1996).

Metal (Me) sulfide neoformation

$${\text{H}}_{2}{\text{S}+\text{Me}}^{2+}\to \text{MeS}\left(\text{precipitated}\right)+2{\text{H}}^{+}$$ (1)

Sulfate reduction coupled to glycerol oxidation at low pH

$$4{\text{C}}_{3}{\text{H}}_{8}{\text{O}}_{3}+7{\text{SO}}_{4}^{2-}+14{\text{H}}^{+}\to 12{\text{CO}}_{2}+7{\text{H}}_{2}{\text{S}+16\text{H}}_{2}\text{O}$$ (2)

Bioremediation of AMD through enhancement of the activity of sulfidogenic microorganisms, predominantly SRB, has been investigated since at least the late 1960s (Tuttle et al., 1969; Bilgin et al., 2007; McCullough and Lund, 2011; Méndez et al., 2021). The strategies for enhancement mainly focused on supplementation with organic carbon, either as defined compounds such as lactate (Luptáková et al., 2016), pyruvate (Calabrese and Bissonnette, 1990), ethanol (Martin et al., 2003) or as complex sources like manure, whey, sawdust and other industry byproducts (Christensen et al., 1996; Castro et al., 1999; Chang et al., 2000; Hao et al., 2014; Sampaio et al., 2020). This was investigated both at field sites (Koschorreck et al., 2007; McCullough et al., 2008) and in more controlled laboratory settings, using in some cases the microbial communities present in the metal-contaminated material (Greets et al., 2006) or mixed external environmental samples as additional microbial seed material (Barbosa et al., 2014; Briones-Gallardo et al., 2022). However, biosulfidogenesis has also been shown to establish naturally in anoxic parts of AMD environments, both in AMD sediments (Sánchez-Andrea et al., 2012; Falagán et al., 2015) and in the deep part of the water column of several permanently stratified (meromictic) APLs (Falagán et al., 2014; Sánchez-España et al., 2020a; van der Graaf et al., 2020), where it was linked to the natural attenuation of extreme conditions. For example, the high relative abundance of sequences affiliated to the genus *Desulfomonile* from the monimolimnion of the eutrophic Brunita pit lake (BR) (La Unión, Murcia) (Sánchez-España et al., 2020a) and the oligotrophic Filón Centro pit lake (FC) (Tharsis, Huelva) (van der Graaf et al., 2020) coincided with an increase in pH and the near-complete removal of Cu from the water column.

In the abovementioned studies, the pH increase and metal removal was more pronounced in the eutrophic lake (BR) than in the oligotrophic lakes (FC, GU). Although this might be related to the presence of carbonate minerals (i.e., chemically-driven abiotic neutralization), the eutrophic conditions (Sánchez-España et al., 2020a) enable extensive algal growth in the upper layer, providing organic carbon to the microbial community throughout the water column, including SRB. Additionally, the biomass by itself might contribute to the metal immobilization through bioassimilation and bioaccumulation, as for example via phosphatase generation of phosphate and metal intake (Macaskie, 1990) or the formation of intracellular mineral precipitates accumulating the pollutants (Mann et al., 1987). This suggests that local differences in the geological and physicochemical characteristics of APL affect the establishment of biosulfidogenesis, and that supplementation with organic carbon might not always be required to trigger bioremediation. From an applied perspective, this indicates that *in-situ* treatment strategies based on biosulfidogenesis must be site-specific.

The goals of the experiment were 1) to evaluate the response of oligotrophic and eutrophic systems to carbon amendment, 2) to investigate the chemical effects and mineral products of SRB activity in these systems, and 3) to evaluate the potential of glycerol amendment to remediate acidic pit lakes and AMD systems via biosulfidogenesis. Glycerol has been previously reported to be an appropriate carbon source for sulfate-reducing bacteria (Qatibi et al., 1991; Santos et al., 2018). In addition glycerol is non-toxic for the environment, easily available in the market and has lower price in comparison to organic substrates mentioned above. Finally, from an operational viewpoint the direct injection of glycerol to the deep SRB-rich layers of APL would be relatively easy to carry out. A better understanding of abiotic and biological factors shaping the biogeochemical evolution of AMD-affected areas will support the design of improved treatment strategies, also providing a unique insight into the biogeochemical dynamics of low temperature, oxic and anoxic, highly acidic systems.

## 2 Materials and methods

### 2.1 Site description and water/sediment sampling procedures

Three meromictic acidic pit lakes (FC, GU, BR) and one acidic stream (TI). The Tharsis mines are among the westernmost mines in the Iberian Pyrite Belt mining district, located in the South of the Iberian Peninsula (Huelva, Spain). Filón Centro (37°35′27.4″N, 7°07′27.5″W) was extensively mined for copper and pyrite from the XIX century until its’ final closure in 1986. Another example of IPB considered in this study was Guadiana Pit lake (37°36′54.8″N, 7°17′37.4″W) from Herrerías Mining complex, located 18 km to the West of Tharsis mines (Sánchez-España et al., 2013; Sánchez-España et al., 2020b). As a model of acidic stream sediments at oxygenic conditions, the Tintillo acidic stream was selected (37°42′44.7″N, 6°37′41.7″W). This acidic creek is formed by leachates from waste-rock piles and tailings ponds situated in the surroundings of the Corta Atalaya open pit at Rio Tinto mine (Sánchez-España et al., 2005; Sánchez-España et al., 2007).

Finally, Brunita open pit (37°36′1″ N, 0°53′23″ W) was selected as an example of meromictic eutrophic lake (Sánchez-España et al., 2020a). Located 6 km to the East of Cartagena (Murcia) in the SE Spain, Brunita mine was one of the most important in the La Unión-Sierra de Cartagena mining district (Oen et al., 1975).

For this experiment, mixolimnetic water was sampled in polyethylene bottles and sediment was directly gathered with a shovel, both from the shallow shore area. Monimolimnetic anoxic sediment was collected using a gravity corer (Uwitec, Mondsee, Austria) and the water from the same depth was obtained using a 5 L Van Dorn sampling bottle (KC, Silkeborg, Denmark). Both water and sediment were stored in hermetic containers until arrival at the University of the Basque Country (UPV/EHU). Tintillo acidic stream sample was gathered several meters downstream from the source in the base of a waste-rock pile. Autochthonous biomass (benthic green algae and streamers growing above the sediments) was added to the sediment and stored in the dark in a sealed container until arrival to the lab.

### 2.2 Incubation column setup

IC allow the study of water, sediment and indigenous microbial communities and its response to the variation of biogeochemical parameters (e.g., nutrient or organic substrate addition) and a detailed continuous monitoring of this evolution, avoiding the need for multiple resource-intensive field campaigns. The use of these model ecosystems allows comparing the dynamics at different depths within the same lake (e.g., IC built with mixolimnetic vs*.* monimolimnetic sediment and water) or between lakes i.e., different microbial communities, initial pH, ORP (i.e., oxic top layer, mixolimnion vs*.* bottom anoxic monimolimnion) and chemical composition of the water can be contrasted.

**FIGURE 1:**
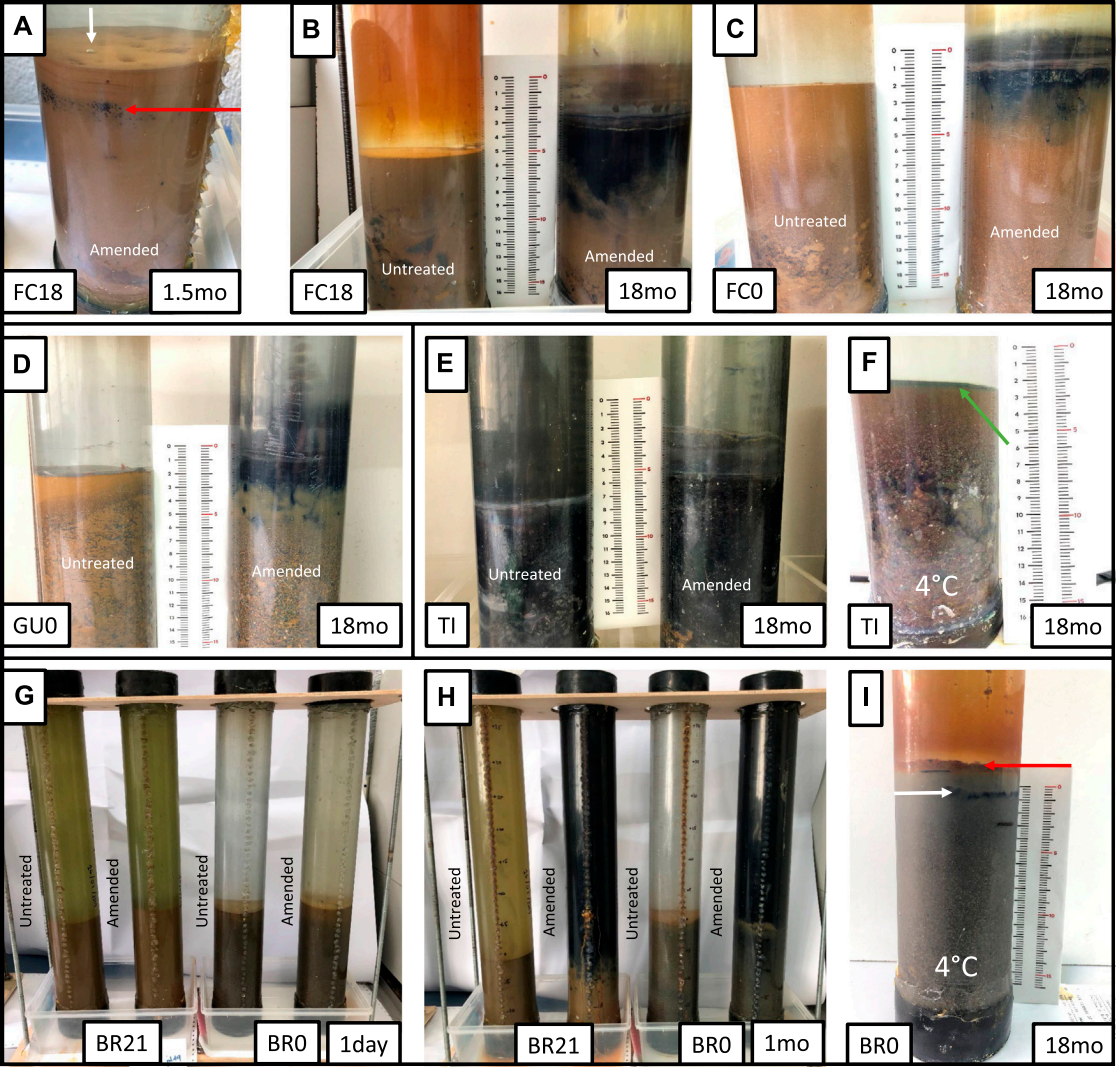
Visual evolution in selected incubation columns at different incubation periods (right bottom corner in months). **(A)** The onset of black layer development (red arrow) and gas bubbles (white arrow) in the sediment of glycerol-amended IC from Filón Centro 18 m (monimolimnion) **(B)** the final state (18 months) of glycerol-amended (right) and untreated (left) ICs from Filón Centro 18 m (monimolimnion) **(C)** Filón Centro 0 m (mixolimnion) and **(D)** Guadiana 0 m (mixolimnion). **(E)** Tintillo acidic stream evolved sediment (18 months) **(F)** cool-stored Tintillo IC remained green (green arrow) after 18 months of incubation. **(G,H)** A single month evolution observed in Brunita 21 m (monimolimnion) and Brunita 0 m (mixolimnion) amended ICs, contrasting with unamended ICs all stored at room temperature. **(I)** Limited changes of cool-stored Brunita 0 m (mixolimnion) IC (18 months) developing a top red layer (red arrow) and very thin black layer (white arrow).

Eighteen Winogradsky-type incubation columns were built using transparent polycarbonate tubes (Uwitec, Mondsee, Austria), which were cut to the length of 60 cm (9 cm in diameter, 3.80 L in volume) and capped with rubber stoppers (Figure 1; Ilin et al., 2021). All cylinders were pre-perforated with hole size of 3 mm every 1 cm and sealed with acetic silicone. These ports allowed a periodic sampling of small volumes of water and sediment at different layers within the columns using hypodermic needles and disposable sterile syringes without noticeable alteration of the incubation process.

The sediments, initially containing detrital quartz, phyllosilicates, minor K-feldspar, rutile, detrital sulfides and (oxy)hydroxysulfates (not shown), were transferred to the IC and then filled up with the lake water from the corresponding depth, leaving no air bubbles, and sealed tight with rubber stoppers. The volumetric ratio of sediment to water was about 1:2 (GU0, TI, BR21 & BR0) or 1:3 (FC18 & FC0) depending on the amount of available solid phase. At the onset of the experiments, the IC were homogenized by vigorous shaking, so that all columns were originally homogeneous, and therefore the vertical gradients would develop as a consequence of microbial activity and biogeochemical processes.

The incubations were performed in dark conditions and three equivalent columns were built for each of the six samples: FC (0 m), FC (18 m), GU (0 m), TI (0 m) and BR (0 m) and BR (21 m). Two columns of each set were incubated at room temperature with and without amendment by addition of 18 mM of glycerol (C_3_H_8_O_3_; Laboratorios Pharma&Go, S.A.) simulating natural and biostimulated microbial growth. Finally, for all sites, a third column was stored at 4°C to minimize biological activity and use it as a control.

### 2.3 Evolution of physicochemical parameters and nutrient concentration

Throughout the incubation period, regular visual examination, geochemical monitoring and water and sediment sampling were performed. The depth 0 cm was always adjusted for reference to the water-sediment interface accounting for early diagenetic changes (e.g., compaction), so that water column is shown with positive values, while sediment has negative values. The pH was measured using a H138 Hach compact pH meter capable of measuring pH in a single drop with an error of ±0.1 pH point according to the manufacturer. The pH sonde was two-point calibrated with 4.0 and 7.0 pH buffers before measurements. A combined pH & ORP YSI Pro1020 probe was used for ORP monitoring, though with less periodicity given the greater volume of sample needed (3 ml per each measuring point). Previously to the regular measurements, HI 7021 240 mV ORP solution (Hanna Instruments, Woonsocket, United States) was used for ORP calibration. The accuracy for ORP measurements is estimated at ±20 mV by the manufacturer.

The evolution of nutrient concentration in the sediment-water interface (low O_2_ or anoxic) and top water (higher O_2_ content) was studied by UV-VIS spectrophotometry using a DR 2800 Hach (Hach, Düsseldorf, Germany) instrument and specific cuvette tests LCK 304 Ammonium (indophenol blue method; Confidence Interval, CI: ±0.01 mg/L as N-NH_4_^+^), LCK 339 Nitrate (4-nitro-2.6-dimethylphenol method; CI: ±0.01 mg/L as N-NO_3_^−^), LCK 349 Phosphate (phosphormolybdenum blue method; CI: ±0.01 mg/L as P-PO_4_^3−^). As an indirect indicator of microbial respiration, dissolved CO_2_ was measured using LCK 388 cuvette tests, which measures total inorganic carbon as carbon dioxide. LCK 653 Sulfide cuvettes (CI: ±0.057 mg/L as S^2−^) based on dimethyl-p-phenylenediamine reaction with dissolved S^2-^ were used for tracking H_2_S evolution.

### 2.4 Geochemical analyses

The geochemical composition of the different IC was determined at different points of the water column at the beginning and at the end (i.e., after 18 months) of the incubations. For each sampling point, 60 ml were extracted through silicone-sealed ports using sterile disposable syringes and filtered through 0.45 µm nitrocellulose membrane filters. The filtrate was immediately acidified with 10 drops of 1M HCl and stored in polypropylene bottles at 4°C. Then it was analyzed by inductively coupled plasma-atomic emission spectrometry (ICP-AES: Fe, Cu, Zn and SO_4_^2−^) using an Varian Vista MPX (Varian Medical Systems, CA, United States) at IGME-CSIC laboratories. For trace elements (e.g., As and Cd), inductively coupled plasma-mass spectrometry (ICP-MS) was performed using a Agilent 7500ce (Agilent Technologies, CA, United States) instruments. The accuracy and precision considering the whole analytical procedures were verified with certified geochemical reference standards such as SRM 1643 (trace elements in water, NIST), APG 4073 (trace metals in waste water, APG), internal samples of industrial mine effluents and control samples of the Water Research Centre’s AQUACHECK international program, obtaining a close agreement with certified values for all metals. The detection limits in the pit-lake and IC waters studied were <1 mg/L for major elements, and <1 μg/L for trace elements (1 μg/L for Zn; 0.4 µg/L for As; 0.5 μg/L for Cd; 0.2 μg/L Cu). Considering the high concentration of reported ions in the ICs compared to the detection limits, the confidence intervals were not plotted.

### 2.5 Mineral identification

Mineralogical identification of suspended particles and sediments at different intervals was performed by different techniques at SGIker analytical services (UPV/EHU). X-ray Diffraction (XRD) was carried out on a PANalytical X’Pert Pro Malvern Panalytical, Almelo, Netherlands diffractometer and interpreted using High Score X’Pert software pack. In order to prevent the artificial formation of minerals (e.g., salts) during sample preparation, suspended particulate matter and sediment samples were washed with MilliQ water in several cycles (repeated centrifugation at 4,400 rpm, Eppendorf 5702 or 13,200 rpm, Eppendorf 5415D) (Eppendorf, Hamburg, Germany), liquid phase elimination, water addition and re-suspension), all performed in the metal-free ISO seven clean room. These samples were mounted on 25 mm C (graphite) stub, subjected to plasma cleaning and coated with C layer. These stubs were examined under a Scanning Electron Microscope (SEM) with field emission source JEOL JSM-7000F (JEOL, Tokyo, Japan) (10 mm working distance, 20 kV, 1 nA) coupled with Oxford INCA 350 (Oxford Instruments, Abindon, United Kingdom) energy dispersive X-ray spectroscopy (EDX) for semi-quantitative point chemical analysis.

### 2.6 Cryo X-ray imaging

The synchrotron based cryo Transmission soft X-ray microscopy (TXM) allows high-resolution image acquisition of unstained and intact biological samples in its almost native state (Kördel et al., 2020). Sediment samples were directly suspended in N_2_ deoxygenized water, allowing the biggest particles to decant during 5–15 min, after which a 2 µl of supernatant were transferred onto Au Quantifoil G200F1 R 2/2 grids pretreated with 1.5 µl of Au 100 nm bits’ suspension. After samples were vitrified following ethane plunge freezing protocol at Leica EM GP2 vitrobot (20°C, humidity 90% and blotting time 3–6 s) were immediately stored in LN_2_. Each grid was screened under Zeiss AxioS1 (Carl Zeiss AG, Oberkochen, Germany) optical light microscope and Linkam CMS196 (Linkam Scientific Instruments, Surrey, United Kingdom) cooling stage at Alba microbiological laboratory.

A series of two-dimensional images were acquired at Zeiss Transmission X-ray Microscope at the Mistral beamline of Alba synchrotron (Sorrentino et al., 2015). From the bending magnet polychromatic radiation, specific energies were selected using a variable line spacing plane grating monochromator incident on the sample tuned at a certain energy, at which particular elements of interest are highly absorbing compared to the surrounding elements. Magnified images of the sample produced by a Fresnel Zone Plate objective lens were acquired on a PIXIS-XO (Teledyne Princeton Instruments, NJ, United States) CCD detector.

To delimitate biological features, the image was firstly acquired at 520 eV (in so called “water window” energy range), localized below O K-edge, but above both C and N K-edges which make up the main contrast in the image. For further investigation of these precipitates, the energy was raised to Fe and Zn absorption edges. The Fe L-edge is a range where the core electrons from 2p orbital are excited to partially filled 3d orbital, creating a very characteristic absorption feature (van der Laan and Figueroa, 2014; experimental spectra for Fe particle can be found in Supplementary Figure S1). For each element an image at pre-edge (below the absorption peak) and at the L_3_ peak maxima were obtained.

### 2.7 Microbial community analysis

**FIGURE 2:**
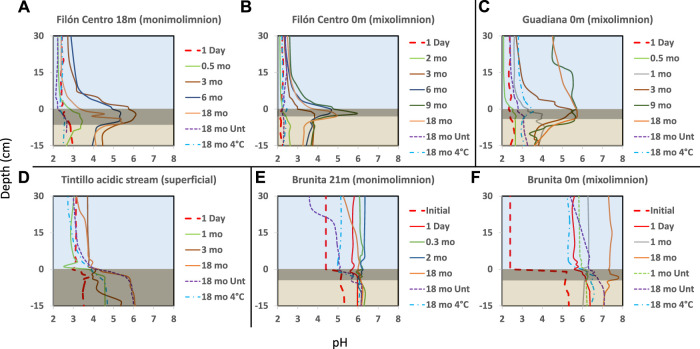
Depth profiles of pH (*x*-axis) throughout the water and sediment (*y*-axis represents depth) in the incubation columns built with sediments from the Filón Centro **(A)** monimolimnion (FC18) and **(B)** mixolimnion (FC0) **(C)** Guadiana mixolimnion (GU0) **(D)** Tintillo stream (TI) and **(E)** the Brunita pit lake monimolimnion (BR21) and **(F)** mixolimnion (BR0). Blue area shows the water column, dark rectangle represents black sediment (gly-amended column) and brownish the least affected sediment column. The line type represents the different treatments: Dashed untreated; Solid: glycerol amended; Dash-dotted: cool-stored (incubated at 4°C). The thick red dashed line represents the initial pH at the beginning of the experiment. Zero depth is bound to the sediment-water interface.

To investigate the effect of glycerol amendment on the final microbial community composition in the different IC, sediments were sampled for 16S rRNA gene amplicon sequencing at the end of incubation (18 months). Sampling depths were selected based on the occurrence of pronounced alkalinization and visual criteria, e.g., the appearance of dark blackish layers. Subsequently, the same depths were sampled in unamended IC, and cool-stored (4°C) columns for comparison. In addition, to investigate whether the pH gradients observed in the sediments of glycerol-amended FC0, FC18, and GU0 IC (Figures 2A–C) corresponded to differences in relative abundance of particular (sulfidogenic) taxa, multiple depths were sampled along the pH gradient in the sediments.

Sediment sampling was carried out at room temperature using sterile hypodermic needles (18G × 1/2”) and disposable syringes (25 ml). IC ports were sterilized before perforation. The syringes containing 25 ml samples were immediately sealed with silicone to avoid oxidation and stored at 4°C. The samples were transported to the University of Wageningen (WUR) in a Styrofoam box at controlled temperature of 2–8°C.

Samples in the sealed syringes were processed at WUR within 30 h after sampling at UPV/EHU. Contents from each syringe were transferred to 15 ml Falcon tubes and centrifuged (4°C, 20 min 4,700 rpm, 4,120 × g). The supernatant was removed and pellets were separated into triplicates (technical replicates), and transferred to the sterile bead tubes from the FastDNA Spin Kit for Soil (MP Biomedicals, OH, United States). Bead tubes were frozen until further processing. DNA was extracted according to manufacturer’s instructions. DNA concentrations were measured using a Qubit 2.0 fluorometer (Life Technologies, Darmstadt, Germany) with the Qubit dsDNA BR assay kit. PCR amplification and library preparation was performed as described in van der Graaf et al. (2020). PCR amplicons were sequenced with the Illumina HiSeq 150 bp paired-end read sequencing (Novogene, Beijing, China).

Paired-end amplicon sequences were processed using NG-Tax 2.0 on the Galaxy platform https://ngtax.systemsbiology.nl (Poncheewin et al., 2020). Sequences were clustered into Amplicon Sequence Variants (ASVs) using a *de novo* clustering approach, and the following default settings: forward and reverse read length 70 nt, ratio ESV abundance 2, classify ratio 0.8, minimum percentage threshold 0.01, identity level 100%, error correction of one mismatch. The filtered reads were demultiplexed per sample, keeping only reads with perfectly matching barcodes. Taxonomic assignment of the ASV’s was done using SILVA SSU rRNA reference database v138 (Quast et al., 2013; Yilmaz et al., 2014). Results were exported as a.biom1 file for further analysis with R (R Core Team, 2021) in RStudio, using the phyloseq (McMurdie and Holmes, 2013), microbiome (Lahti and Shetty, 2017), ggplot2 (Wickham, 2008), ggpubr (Kassambara, 2020) and dplyr packages in the tidyverse (Wickham et al., 2019). R scripts and input files can be accessed at https://github.com/mibwurrepo/Ilin-et-al-2022. The sequences have been deposited in European Nucleotide Archive (ENA) at EMBL-EBI under accession number PRJEB52361 (secondary accession number ERA12717712).

## 3 Results

### 3.1 Development of pH and redox gradients during incubation

**FIGURE 3:**
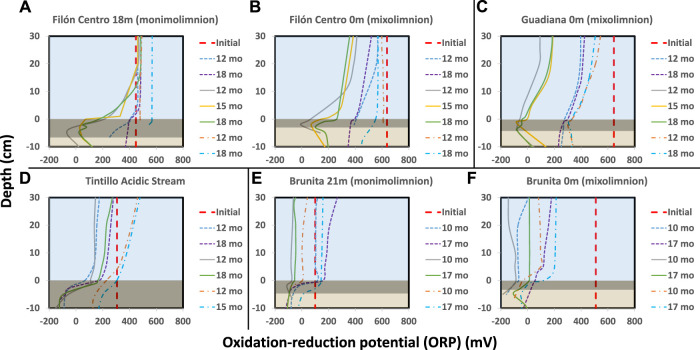
ORP evolution over time in the different IC. **(A)** FC 18 m **(B)** FC 0 m **(C)** GU 0 m **(D)** TI stream **(E)** BR 21 m **(F)** BR 0 m. Thick red dashed line shows the initial ORP at the beginning of incubation; thin dashed line marks the evolution in untreated columns, solid line in glycerol amended and dash-dotted cool-stored IC. Blue area shows the water column, dark rectangle black sediment (gly-amended column) and brownish the least affected sediment column.

The effect of glycerol amendment was visually monitored, as well as the evolution of physicochemical parameters (pH, ORP), in the IC built from oligotrophic (FC, GU), eutrophic (BR) APLs and the oligotrophic AMD-affected stream (TI). The pH alkalization occurred in the majority of IC from all the different sites, where clear differences in the development of layers within the water column and sediments (Figure 1), timing and shape of pH/ORP profiles (Figures 2, 3) in response to glycerol amendment could be observed.

#### 3.1.1 Filón Centro

In amended IC built with sediments from 18 m depth from the oligotrophic FC (FC18), the visual evolution became noticeable after only 1–1.5 months (hereafter mo) (Figure 1A, Supplementary Figure S2), while untreated and cool-stored IC remained visually unchanged during the 18 months incubation period (Figure 1B). In the amended IC, the sediment started darkening in small pockets of up to 1.5 mm in diameter at depths of 2.5 cm (Figure 1A, red arrow). Throughout the incubation period, these pockets kept expanding in size, forming a continuous black layer of 0.75 cm by 1.5 months and 6.5 cm at the end of the incubation (Figures 1A,B; Supplementary Figure S2). The pH (Figure 2A) and ORP (Figure 3A) dynamics were restricted to the first 10 cm of the sediment, with the strongest pH increase at -4 cm depth. After only 14 days of incubation, the peak value of pH increased from 2.8 to 3.4, and kept increasing to the maximum of 6.1 (3 months), also showing outstanding acidity neutralization in the first 7 cm of the water column (pH 3.2–5.7). The ORP in the amended IC decreased from 448 mV to −62 mV (12 months) and slightly increased again to 27 mV (15 months; Figure 3A), coinciding with progressive acidification. After 1 year, a separation of the broad pH peak was observed with the first maximum around −2 cm (pH 5.0; ORP 52 mV) and the second at −5 cm (pH 5.4; ORP 23 mV). The untreated and cool-stored ICs showed oxidation trend with ORP values of 395 and 568 mV at the end of incubation respectively.

The IC built with mixolimnetic sediment and water from the same pit lake (FC0) also showed no visual or pH evolution (Figures 1C, 2B) in untreated and cool-stored IC, while after 18 months ORP decreased from an initial range of 637 to 345 mV (sediment) and 430–400 mV (water) to 568 mV (water) and 460 mV (sediment), respectively (Figure 3B). In contrast to FC18, the amended IC had a longer lag phase taking a larger period to achieve the maximum pH values, along with a smaller affected area. The sediment darkening became visible after about 5 months expanding from the sediment interface downwards (Supplementary Figure S3). The thickness of this darker sediment increased from 0.3 cm (5 months) to 3 cm (18 months) (Figure 1C, Supplementary Figure S3). The peak pH values showed limited increase during the first 2 months (2.2–2.6), reaching pH 5.9 after 9 months, with subsequent decrease to pH 5.3 (ORP −8.9 mV, 12 months) and pH 4.9 (ORP 101 mV in sediment and 388 mV in water column) at the end of incubation (18 months).

#### 3.1.2 Guadiana

Mixolimnetic Guadiana pit lake IC (GU0) showed similar dynamics in untreated and 4°C-stored columns, exhibiting no visual changes in the sediment, while the water column gradually acquired a reddish tint in the upper part in the advanced stage (Figure 1D; Supplementary Figure S4). The pH in the untreated column gradually increased from 2.3 to pH 3.3 (Figure 2C) and ORP decrease from 645 mV to 359–539 mV in water (Figure 3C) and 300 mV in the sediment (max values in untreated IC); pH 3.5 and ORP down to 276–403 mV in water and 230 mV in the sediment in 4°C IC. Sediment in glycerol-amended IC began darkening after 2 months, gradually extending in thickness to the first −4 cm (Figure 1D). The pH progressively increased in the sediment from 2.3 to peak values 4.0 in the first month and gradually extending to the overlaying water column. Dense black turbidity appeared after 9 months of incubation related to the pH raise to 5.7 in the first +15 cm, abruptly decreasing to pH 4.5 above that point. That sharp transit from the pH of 5.7 to 4.5 in few cm above +15 cm height had been flattening in the last 3 months of incubation. The pH showed little variation between 9 and 18 months, with the maximum value around 5.7 at −3 cm and ORP as low as −40 to −82 mV in the sediment.

#### 3.1.3 Tintillo

Regarding the Tintillo acidic stream IC (TI), although the stream by itself is oligotrophic, the collected sediment was admixed with autochthonous photosynthetic benthic microalgae growing in the river bed (Supplementary Figure S5A), providing a high initial input of C, N and P, and thus inducing artificially eutrophicated conditions in these ICs. The cool-stored column acquired a reddish tint, which gradually expanded from the top downwards, but maintained a green colored sediment (Figure 1F, green arrow). However, both untreated and glycerol-amended columns showed noticeable darkening after 4.5–2 months respectively (Figure 1E; Supplementary Figure S5). The pH increase was initially restricted to the sediments, increasing from 3.8 to 5.9 in 9 months in the untreated and 2.5 months in amended ICs (Figure 2D). In the untreated column, the dark turbidity appeared after 4.5 months (gradually ascending until turning completely dark after 12 months) with thin white layer at the water-sediment interface. The pH of the onset of dark turbidity was in the range of 4.4 (milky-white layer) and 3.9 (top point of dark turbidity) at 4 months. ORP in both untreated and amended columns decreased from 307 mV to 162–251 mV (water) and to -124 mV (sediment; Figure 3D), while cool-stored column had a decrease in the sediment (down to 128 mV) and limited increase in the water column (380 mV). Between 12 and 18 months the pH remained almost unchanged, coupled with oxidative ORP trend, more pronounced in the upper part of the water column.

#### 3.1.4 Brunita

In the ICs from Brunita pit lake (BR21 and BR0), the pH developed mostly uniformly throughout the water and sediment, and much faster and to higher values than in the ICs from the other sites (Figures 2E,F). In 1 day, the pH in BR21 increased from 4.4 along the water column and 5.3 in the sediment to 5.7 and 6 respectively (Figure 2E), and achieving pH 6.1–6.3 uniformly distributed along the amended column after only 2 months, coupled with a decrease in ORP from 100 mV to −112 mV (Figure 3E). After that maximum, the pH in the water column began to decrease down to 5.1. A similar evolution was observed in the untreated columns with even lower pH (5.2) and ORP (144 mV) in the sediment-water interface), or 3.7 and 208 mV at 30 cm above this layer. In BR21, dense green-yellowish turbidity appeared on the first day in both the amended and unamended ICs. The IC turned completely black after 1 month (amended) (Figures 1G,H) or 2.5 months (untreated). The black coloration in the sediment in the amended IC appeared in the first week as patchy growth style (Supplementary Figure S6), and just 4 days later displayed a 1.2 cm-thick continuous layer, expanding to 5 cm at the end of the incubation period. In the untreated IC it took 1.5 months for the development of the black layer, extending down to 1.8 cm after 18 months. The cool-stored IC showed no darkening, but instead a thin red layer developed on top of the sediment. In both BR21 and BR0, the pH neutralization began from the very first moment after the column homogenization (Figures 2E,F). In 1 day, the pH in BR21 increased from 4.4 along the water column and 5.3 in the sediment to 5.7 and 6 respectively (Figure 2E), and achieving pH 6.1–6.3 uniformly distributed along the amended column after only 2 months, coupled with a decrease in ORP from 100 mV to −112 mV (Figure 3E). After that maximum, the pH in the water column began to decrease down to 5.1. A similar evolution was observed in the untreated columns with even lower pH/ORP 5.2 and 144 mV (sediment-water interface), 3.7 and 208 mV (+30 cm).

In contrast, the mixolimnetic column (BR0) (Figures 1G–I; Supplementary Figure S7) had a more intense neutralization in the first day from pH 2.4 to 5.8 in the water and from 5.1 to 6.3 in the sediment. (Figure 2F). From that point, the alkalinization in the amended column gradually increased in water and sediment to pH 6.8 and −90 mV and pH 6.8 and −184 mV after 10 months, with a maximum at −4 cm in the sediment (Figure 3F). The evolution in the untreated column was more limited, with the pH rising in the sediment to 6.3 and −180 mV. The cool-stored column showed a discontinuous thin dark layer after 18 months (Figure 1I). This was not observed for any other cool-stored IC. The IC had a slight increase of pH to 6.6 in the sediment and to pH 5.3 in the water column, but showing substantial ORP reduction from the initial 510 mV in both water (90 mV at 10 months; 200 mV at 17 months) and sediment (−106 mV at 10 months; −50 mV at 17 months). Both ICs with and without amendment showed an oxidative trend between 12 and 18 months, although unlike the rest of IC, the pH kept rising with a maximum at 7.7.

### 3.2 Evolution of CO_2_ and nutrient concentration

**FIGURE 4:**
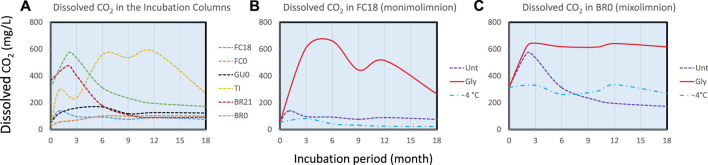
**(A)** Dissolved CO_2_ at water-sediment interface (between 0 and +1 cm) over time in the untreated ICs from different sites. Detailed comparison among ICs with different treatment is shown for **(B)** Filón Centro 18 m (monimolimnion) and **(C)** Brunita 0 m mixolimnetic IC. Dashed line represents the evolution in untreated columns (unt), solid in glycerol amended (gly) and dash-dotted cool-stored (4°C) IC.

Dissolved CO_2_ was measured at different stages of the incubations as an estimation of the intensity of the bacterial metabolism in each column at the water-sediment interface (Figure 4A). The comparison among untreated IC (Figure 4A) shows initial peak of CO_2_ accumulation in BR21 and BR0 after 2 months of incubation, a small peak after 1 month in FC18 and slow accumulation in GU0. TI showed a progressive accumulation gradually decreasing towards the end of incubation. Figures 4B,C show the response of each of three types of treatment in FC18 and BR0 ICs. In both cases, CO_2_ generation was considerably higher in glycerol-amended columns, which was more pronounced in FC18 (similar maximum concentrations of 661 and 644 mg/L CO_2_ in FC18 and BR0, respectively, but over longer period for the latter). The carbon dioxide concentration decreased towards the top of the water column, confirming that the production of this gas was mainly generated at the water-sediment interface where bacterial activity was most intense (e.g., TI 559 mg/L and 119 mg/L CO_2_ or FC0 110 mg/L and 55.5 mg/L CO_2_ after 6 months at bottom and top, respectively).

**FIGURE 5:**
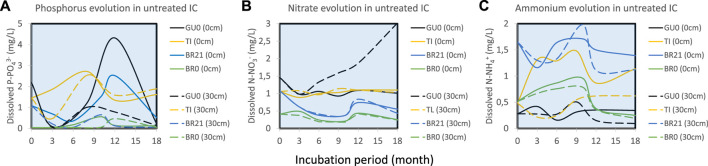
The evolution of **(A)** phosphorus and inorganic nitrogen, as **(B)** nitrate and **(C)** ammonium, in the different untreated IC.

Phosphorus concentrations, measured as P-PO_4_^3−^ (Figure 5A), generally decreased in ICs from FC, GU and BR, during the first 3–4 months of incubation at both sediment-water interface and on the top of the water column, followed by a considerable release at water-sediment interface at the advanced stage (9–12 months). In TI, however, phosphorus concentration increased during the first 9 months, after which slowly decreased in the advanced stage.

Inorganic nitrogen was measured as both N-NO_3_^−^ (Figure 5B) and N-NH_4_^+^ (Figure 5C). The former tended to be more abundant at the top of the water column, while ammonium was dominant at the water-sediment interface. FC18, FC0 and GU0 showed similar gradually ascending trend of nitrate at the top of the water column, while BR21 and BR0 had an initial decrease in the first 9 months, followed by an increase with a peak at 12 months of incubation (Figure 5B). TI barely showed any evolution of nitrate, but revealed a considerable formation of NH_4_^+^ at the water-sediment interface. Limited ammonium release was also detected in the rest of IC at the early stage of incubation.

### 3.3 Geochemical evolution during the incubation period

**FIGURE 6:**
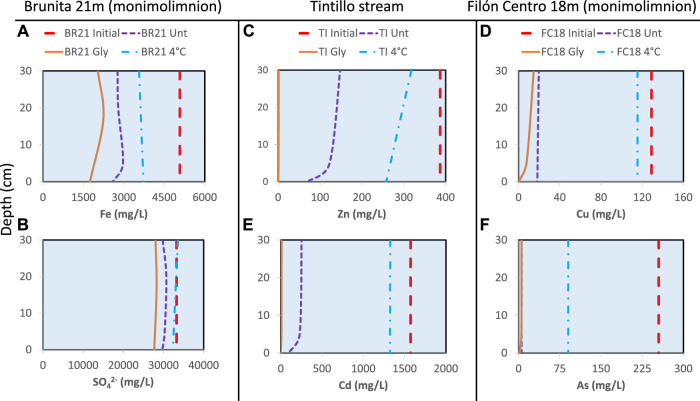
Metal concentration profiles obtained at different points of the water column overlaying the sediment. **(A)** Iron **(B)** sulfate (SO_4_^2−^) **(C)** zinc **(D)** copper **(E)** cadmium **(F)** arsenic. The concentration at the beginning of incubation (red dashed line) is compared to the concentration of these elements after 18 months of incubation with different treatment: untreated (dashed violet line), amended (solid orange line) and 4°C (dash-dotted blue line).

Chemical analysis of water column samples showed the geochemical effect of the different treatments in each set of incubation columns after 18 months of incubation. Iron was among the major elements in all of the studied systems. The decrease of dissolved Fe was detected at the top of the water column of untreated and amended IC, while at the sediment-water interface it had either decreased in both BR21 and BR0 (Figure 6A) or increased in the rest of the ICs, being more pronounced in amended columns as or instance up to 4-fold increase in TI or 11-fold increase in GU0 (Supplementary Figure S8). A similar pattern was observed for dissolved SO_4_^2−^, where the net decrease was only observed in Brunita ICs (Figure 6B; Supplementary Figure S9). FC18 showed depletion in sulfate in the upper part of the water column, whereas towards the sediment-water interface the dissolved Fe increased compared to the beginning of the incubation. For both ions, the dynamics were more significant in amended IC, while cool-stored remained less affected.

Zn and Cu showed a non-conservative behavior, with almost complete removal in the amended columns (Supplementary Figures S10, S11). In untreated IC these ions decreased toward water-sediment interface ranging from Zn concentrations around 153 mg/L near the top of the water column to 70 mg/L at sediment-water interface in TI untreated column (Figures 6C,D). The only exception was FC0 untreated column, where the decrease was from 15.4 mg/L to 14.9 mg/L and 13.2 mg/L at the interface. BR21 and BR0 showed a complete removal of Cu and Zn in all IC, with the exception of Zn being less affected in cool-stored IC (BR21 decreased from 408 mg/L to 124–119 mg/L and BR0 from 194 mg/L to 46–18 mg/L). Untreated columns FC18, FC0, GU0 and Tl showed lesser metal immobilization compared to glycerol-amended and less effect on 4 °C IC.

Two of the most toxic elements in these systems, Cd and As, also experienced an almost complete removal in all IC from Brunita pit lake (BR21 and BR0), similar to the rest of glycerol-amended IC, being especially low towards the water-sediment interface (Figures 6E,F; Supplementary Figures S12, S13). This tendency was also observed for both Zn and Cd. Untreated IC proved to be less affected, while cool-stored had lower immobilization rate for As and either low decrease or even increase in Cd, the latter detected in both FC18 and FC0.

### 3.4 Mineral formation

**FIGURE 7:**
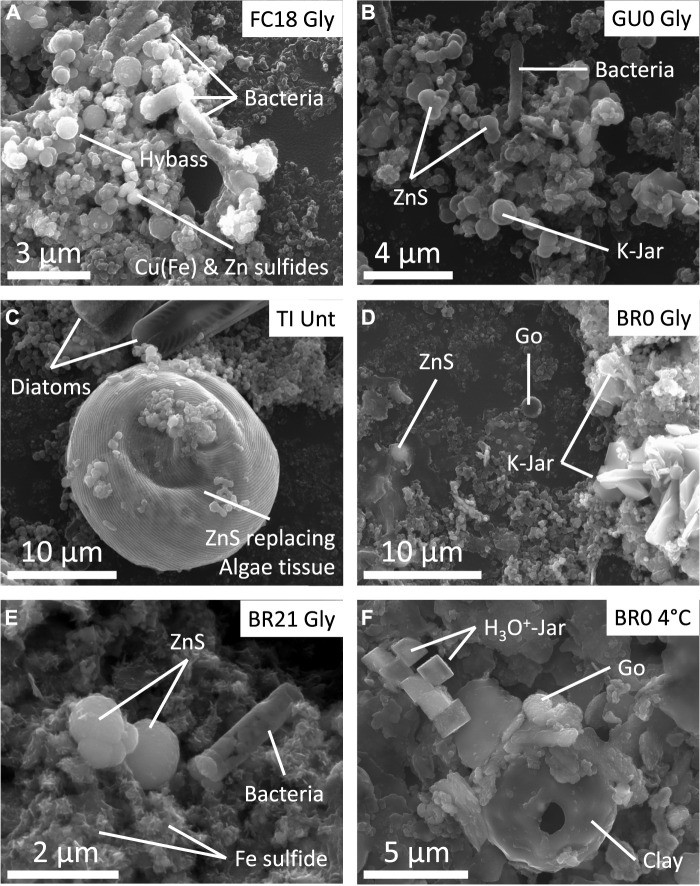
Scanning electron microscope (SEM) micrographs of different samples extracted from IC at different incubation states. **(A)** Abundant fossilized microbial cells surrounded by hydrobasaluminite (Hybass), Zn, Cu and/or Cu-Fe sulfides from the black layer of FC18 (monimolimnion) IC. **(B)** Abundant microbial molds surrounded by K-jarosite, hydrobasaluminite, Cu and Zn sulfides. **(C)** Algae tissue showing a considerable replacement by Zn sulfides and diatoms from TI untreated column. **(D)** Dark turbidity sample from BR0 amended IC, showing the presence of goethite (Go), jarosite (Jar) and Zn sulfide. **(E)** Zn and Fe sulfides, as well as fossilized microbial cells in the sediment sample from BR21 amended IC. **(F)** Fe precipitates (goethite and H_3_O^+^-Jarosite) from BR0 4°C stored IC.

SEM examination showed widespread formation of metal (oxy)hydroxysulfates at the initial stage of incubation. The Fe/S elemental relation and characteristic pincushion shape was coherent with early iron oxyhydroxysulfate schwertmannite (Fe_8_O_8_SO_4_(OH)_6_ * nH_2_O) precipitate, which occasionally fossilized the preexisting microorganisms (Figures 7A,B,E). These particles showed incorporation of As, which is a common feature of this mineral (Regenspurg and Peiffer, 2004; Burton et al., 2009; Burton et al., 2021). A higher Fe/S ratio and spherical, globular or idiomorphic crystals, with or without K, identified other iron particles as Fe hydroxysulfate jarosite (K/H_3_O^+^ Fe_3_(SO_4_)_2_(OH)_6_; Figures 7B–D,F). During incubation, these early precipitates tended to release the sulfate ion, recrystallizing towards Fe oxyhydroxide (likely goethite). At higher pH (>4.0) Al oxyhydrosulfates were detected in all ICs except in Brunita IC (Figure 7A).

In the advanced stage, Zn, Cu and Fe sulfides were detected in the dark turbidity water (Figure 7D) and black layers in sediment (Figures 7A–C,E) in untreated and glycerol-amended IC. The shape of these sulfides was predominantly spherical (setting it apart from irregular shape of detrital sulfides from the pit walls), though in the TI IC they showed a close association with organic tissues and algae filaments, pervasively replacing or completely covering the organic matter preserving the original shape (Figure 7C). No sulfides were detected in the cool-stored ICs, including in the thin dark level that appeared at BR0 column (Figure 1F and Figure 7F). Rather, exclusively Fe (oxy)hydroxysulfates’ formation was observed in this seam.

**FIGURE 8:**
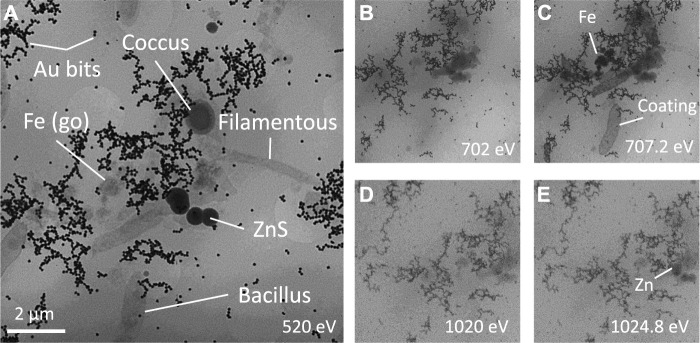
Cryo Transmission X-ray microscopy images at **(A)** water window, Fe **(B)** pre-edge and **(C)** edge, Zn **(D)** pre-edge and **(E)** edge revealing the variety of filamentous, bacilli and coccus bacteria, Fe oxyhydroxide and Zn sulfides from Tintillo glycerol-amended IC.

TXM showed the presence of microorganisms, predominantly rod-shaped cells and less abundant cocci and filamentous cells, coexisting with mineral precipitates (Figure 8A). Because the Fe absorption is changing orders of magnitude between pre- and edge energies, while for all the other elements it is almost the same, the simple comparison of the two TXM images clearly show the presence of abundant Fe precipitates, likely being goethite (Figures 8B,C). Rod-shaped cells showed different degree of Fe coating (Figure 8C), whereas filamentous cells had very little and cocci had no mineral precipitates in direct contact with their membranes. Previous studies by Scanning Transmission Electron Microscopy (STEM) and Electron Energy Loss Spectroscopy (EELS) have demonstrated that these Fe-coatings in anaerobic bacterial cells inhabiting deep layers of acidic pit lakes consist in Fe(II)-SO_4_ ionic complexes adsorbed on carboxylic groups of the cell membrane (Sánchez-España et al., 2018). These coated bacteria were the only ones, that were preserved after the plasma cleaning procedure and under the vacuum conditions of SEM, showing up as external molds (Figures 7A,B,E). Zn L_3_-edge imaging confirms that the dark spherical particle cluster in the central part of Figure 8A appearing in close relation to the microorganisms contains Zn (Figures 8D,E). In this system, the only Zn minerals have proven to be sulfides, allowing us to unambiguously label these minerals as ZnS.

### 3.5 Microbial diversity analysis

After filtering of the 16S rRNA amplicon sequence reads, between 745586 and 1744 remained per sample. In total 1,247 unique ASV’s were detected, representing 417 unique taxa, and between 156 and 19 unique taxa were detected in the individual samples.

**FIGURE 9:**
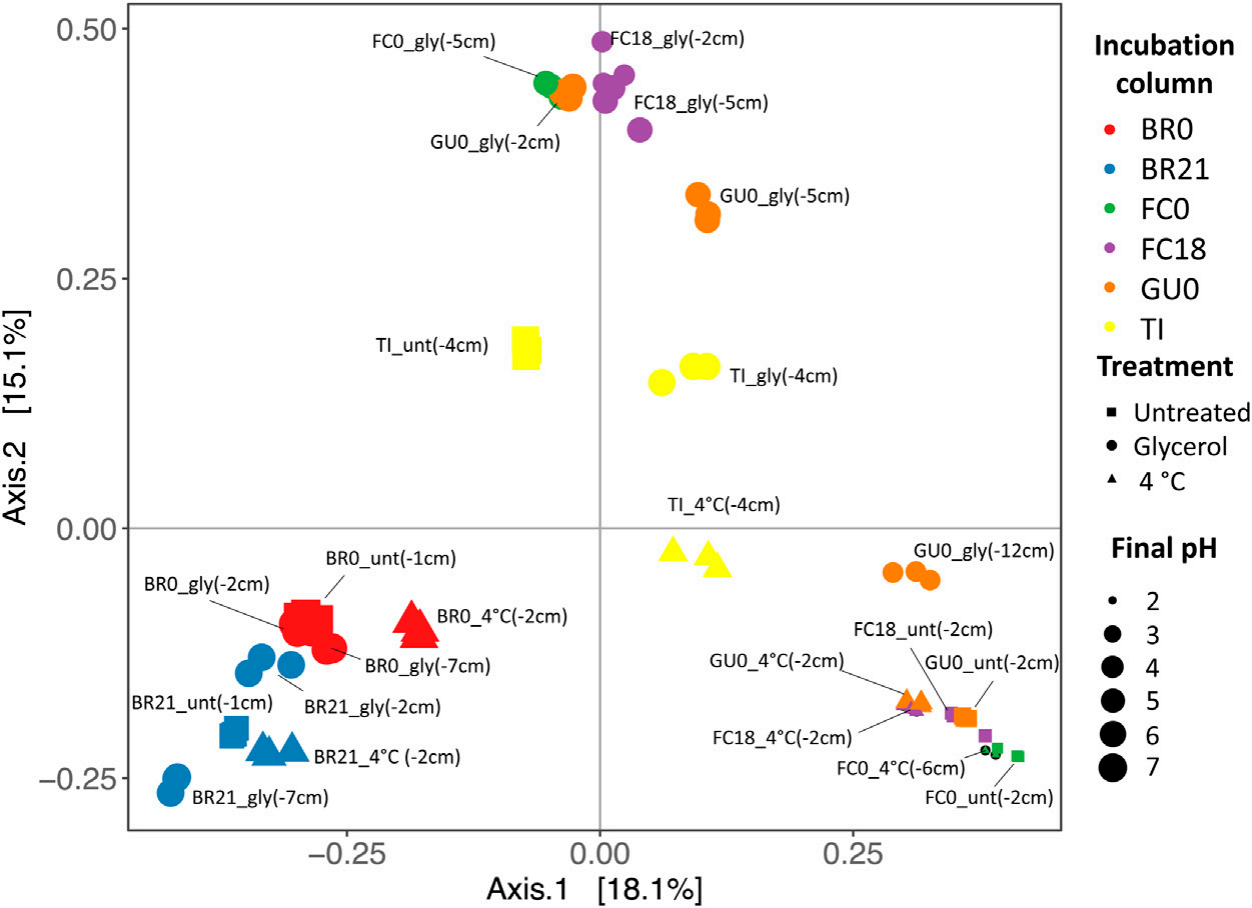
Principal Coordinates Analysis (PCoA) based on the Bray Curtis dissimilarity between samples. Per set of triplicates, the site, treatment and sampling depth (cm) are displayed. Treatment is reflected as gly (glycerol-amended, circles), unt (untreated, squares), 4°C (cool-stored, triangles). Colors represent the original site + depth (red: Brunita monimolimnion (BR0), blue: Brunita mixolimnion (BR21), green: Filón Centro mixolimnion; purple: Filón Centro monimolimnion (FC18), orange: Guadiana mixolimnion (GU0); yellow: Tintillo stream (TI)). The size of the shapes indicated the final pH (2:7, small:large).

Comparison of the microbial diversity between samples (Beta-diversity) showed that BR0 and BR21 were most similar to each other, regardless of treatment, as they clustered away from the other IC (Figure 9). Samples from FC0, FC18, and GU0 grouped in clusters but separated by treatment—one group represented the glycerol-amended IC, while the other group contained the untreated and cool-stored IC. One sample from the glycerol-amended column of GU0 was closer to the second cluster, which was likely related to it being taken from a deeper part of the sediment (12 cm) where no extensive pH neutralization had occurred. The samples from TI did not clearly cluster within any of the three groups described above, but instead formed a separate cluster in between. As could be expected based on the different effect of glycerol-amendment on pH development in BR0 and BR21 compared to the other ICs, the clusters also reflected differences in local pH at the time of sampling—the pH of all Brunita samples was between 6.1 and 7.4, while the samples in the other two groups had a pH between 2.2 and 3.1 at the time of sampling (the unamended and cool-stored IC), or between 4.5 and 5.7 (the glycerol-amended IC).

### 3.6 Microbial community composition

**FIGURE 10:**
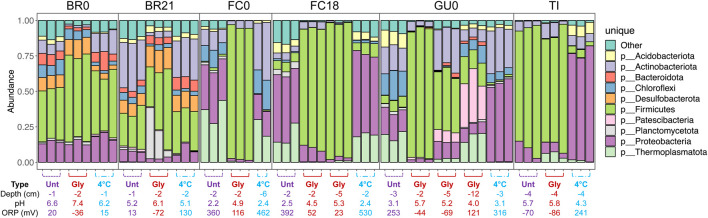
Relative abundance of 16S rRNA gene amplicon sequence reads assigned to the 10 most abundant phyla. For each triplicated sample the depth, pH and ORP are given below.

Comparison of the relative abundance of the top 10 most abundant phyla detected across samples (Figure 10) reflects the grouping of the samples discussed above (Figure 9). In the glycerol-amended IC of FC0, FC18, and GU0, taxa assigned to the phylum *Firmicutes* increased dramatically in relative abundance compared to the unamended and control IC. In the deepest sample (12 cm) from GU0 this increase was not as pronounced, which correlates to the absence of pH increase at this depth (12 cm). In TI, *Firmicutes* were present at high relative abundance in both unamended and glycerol-amended IC, but not in the cool-stored. IC. In IC from BR0 and BR21, *Firmicutes* were present in all columns and did not show as clear an increase in response to glycerol amendment. Several phyla were present in only a subset of the samples. The phylum *Desulfobacterota* was only detected in the IC built from Brunita lake (BR0 and BR21), while *Thermoplasmatota* were detected in IC from FC0, FC18, GU0 and TI. Reads classified to the phyla *Planctomycetota* or *Patescibacteria* were detected only in BR21 and GU0, respectively.

**FIGURE 11:**
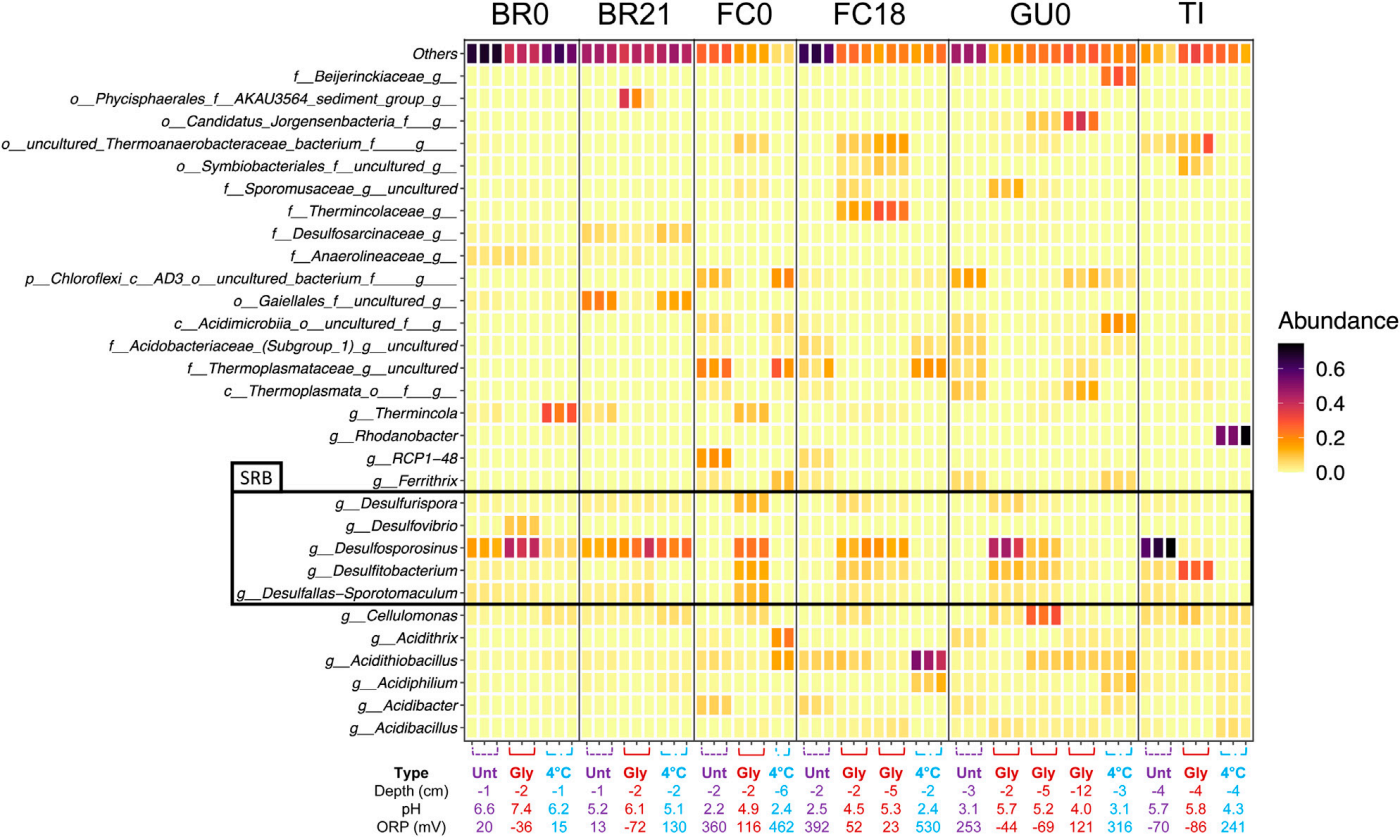
Relative abundance of the 30 most abundant taxa detected across sediment samples in the 16S rRNA gene amplicon sequence reads. Results are grouped per site. Individual replicates are shown and the depth, pH and ORP are given below.

Comparison of the top 30 most abundant taxa detected across samples (Figure 11) shows that in IC built from FC and GU sediments, and to a lesser degree in TI sediments, glycerol-amendment clearly influenced the relative abundance of multiple taxa, including several genera known to contain sulfate-reducing species (Figure 11). Specifically, reads assigned to the genera *Desulfurispora*, *Desulfosporosinus*, *Desulfitobacterium*, which was manually identified as the recently published novel genus *Acididesulfobacillus*, and *Desulfallas-Sporotomaculum* were detected in high relative abundance in the glycerol-amended IC from FC0, FC18, and GU0, while they were not present in high abundance in the corresponding unamended and cool-stored ICs. Although a high relative abundance of known SRB taxa was detected in both the unamended and glycerol-amended IC built from TI sediments, glycerol amendment resulted in a switch from one dominant SRB, *Desulfosporosinus* in the unamended IC, to *Acididesulfobacillus* in the amended IC.

In the IC built from BR0 and BR21 sediments, the impact of glycerol-amendment on the relative abundance of reads assigned to known SRB taxa was not as pronounced as in the IC built from other sediments. The known SRB genus *Desulfosporosinus* was present at high relative abundance in all IC from BR0 and BR21, although its relative abundance increased in the glycerol-amended IC from BR0. Glycerol amendment did appear to increase the relative abundance of reads assigned to the genus *Desulfovibrio*, but only in IC built from BR0 sediments. In addition to *Desulfosporosinus*, reads assigned to *Desulfurispora*, *Acididesulfobacillus*, and *Desulfallas-Sporotomaculum* were detected in the IC from BR0 and BR21, but at low relative abundance.

Glycerol-amendment further appeared to affect the relative abundance of reads assigned to several other taxa (Figure 11). Reads assigned to the genus *Thermincola* were detected in the unamended IC of both BR0 and BR21, as well as in the cool-stored IC of BR0, but were absent of present at less than 1% relative abundance in the glycerol-amended IC from either. The opposite effect was observed in IC from FC0 and FC18 sediments—reads assigned to *Thermincola* were abundant in the glycerol-amended IC, with an additional ASV classified as belonging to the family Thermincolaceae detected at increased relative abundance in the glycerol-amended IC of FC18, while these were not abundant in the unamended and cool-stored IC. Reads assigned to the genus *Cellulomonas* were detected across the sites, but were either only present in the cool-stored IC (BR0 and BR21), the glycerol-amended IC (FC0, FC18, GU0), or in all three IC (TI). The relative abundance of reads assigned to several ASV’s that could not be classified to genus level also appeared to be enhanced by glycerol amendment in FC0, FC18, GU0 and TI IC. These ASV’s represented the family Sporomusaceae and the orders Thermoanaerobacteraceae *bacterium*, *Symbiobacteriales* and “Candidatus Jorgensenbacteria” (Figure 11).

Although some development likely had occurred during incubation at 4°C, the cool-stored IC would be a closer approximation of the microbial community composition at the beginning of the incubations. Except for the IC from BR0 and BR21, no known SRB taxa were detected in the sequenced reads from the cool-stored IC from the other sites. Rather, reads were assigned to genera commonly found in AMD environments such as *Acidithrix, Acidithiobacillus, Acidiphilium, Ferrovum, and Ferrithrix.* Interestingly, the sequenced reads from the cool-stored IC of TI were dominated by *Rhodanobacter*, which was only present in this sample.

## 4 Discussion

### 4.1 Effects of glycerol amendment on bacterial community composition

The incubation columns used in the present study represent useful model ecosystems for the investigation of natural geomicrobial interactions. This experimental approach provides an inexpensive way for testing the response of these complex systems to different treatments, being especially relevant for biotechnological assays. These ICs are also useful to study the microbial dynamics at the water sediment interface in the deep anoxic layers of acidic pit lakes, which are very challenging for direct in-lake studies (Wendt-Potthoff et al., 2012; Diez-Ercilla et al., 2019).

The reported results from the ICs built from oligotrophic sediments (FC0, FC18, GU0) indicated that glycerol amendment is an effective strategy for inducing biosulfidogenesis with the concomitant pH alkalinization and metal precipitation in oligotrophic AMD environments. The role of sulfidogenic microorganisms was supported by the high relative abundance of 16S rRNA gene amplicon sequence reads assigned to known SRB taxa in the glycerol-amended IC from these sites, compared to their absence or low abundance in the corresponding unamended and cool-stored IC. It furthermore confirmed that organic carbon availability is an important factor for the activity and growth of SRB responsible for acidity consumption, whereas phosphorus and nitrogen proved to be less important as limiting nutrient. In agreement with this, glycerol amendment was not required for the establishment of SRB taxa in the IC built with eutrophic sediments from Brunita pit lake (BR0 and BR21) and Tintillo stream (TI), as the abundant presence of algae at the original sampling sites indicated the availability of organic carbon.

The absence of sequenced reads affiliated to known SRB taxa from the cool-stored FC and GU ICs indicated that SRB were not dominant in the sediments used as inocula, corresponding to previous results from the original APL as it was also shown previously for GU (Falagán et al., 2014). In response to glycerol amendment, the SRB taxa *Desulfurispora, Desulfosporosinus, Acididesulfobacillus* and *Desulfallas-Sporotomaculum* were enhanced across the IC from GU0, FC0 and FC18. Their higher abundance at depths with higher pH in FC18 and GU0 further correlates the presence of SRB with an increased pH. The detection of *Desulfosporosinus* was not surprising, as the *Desulfosporosinus* genus comprises several acidophilic or acidotolerant members, with strains isolated from AMD environments (Alazard et al., 2010; Sánchez-Andrea et al., 2015; Panova et al., 2021). Although no acidophilic members from the genera *Desulfurispora* and *Desulfallas-Sporotomaculum* have been officially described, it is worth mentioning that acetotrophic sulfate-reducing consortia developed active biofilms on zeolite and glass beads in batch cultures at initial pH 3 (Campos-Quevedo et al., 2021).

Recently a novel species representing a novel genus of acidophilic SRB was described, *Acididesulfobacillus acetoxydans* (Sánchez-Andrea et al., 2022). In previous studies, sequences belonging to this genus was incorrectly classified as uncultured *Desulfitobacterium* (Sánchez-Andrea et al., 2013) (pH 3.8–6.5, opt 5.0), since both genera branch closely in phylogenetic tree reconstructions. Combined with the fact that *Desulfitobacterium* species are not able to reduce sulfate to perform reductive dehalogenation (Villemur et al., 2006), this raised doubts regarding the correctness of the classification of sequenced reads as *Desulfitobacterium* in this study. Therefore the 27 ASV’s classified as *Desulfitobacterium* were examined in more detail. Closer inspection showed that 26 of the 27 “*Desulfitobacterium”* ASV’s had more than 98% similarity with the 16S rRNA gene sequence of *A. acetoxydans*. The remaining ASV was only detected at 0.1% relative abundance in one of three replicated samples from the glycerol-amended GU0 IC (12 cm) and showed 94.3% similarity, indicating that it indeed represented *Desulfitobacterium*. It was therefore concluded that apart from the one ASV detected in GU0, the reads classified as *Desulfitobacterium* in the present study (according to SILVA SSU rRNA database v138.1) represent *Acididesulfobacillus*, and they are therefore referred to as such.

An important metabolic feature of *A. acetoxydans* is its capacity for complete oxidation of organic acids to CO_2_ (Sánchez-Andrea et al., 2022). This trait is not commonly found in acidophilic SRB. In *Desulfosporosinus* species, acetic acid oxidation has only been recently reported in *D. metallidurans* (Panova et al., 2021) at low amounts, and the only *Desulfurispora* species described so far is not capable of acetic acid oxidation (Kaksonen et al., 2007). Lowering organic acid concentrations in the environment through their complete oxidation was proposed to constitute an important mechanism for *A. acetoxydans* to cope with the organic acid toxicity at low pH. At pH values below their pK_a_ (acetic acid pK_a_ = 4.8), organic acids exist in their protonated form and can diffuse freely over the membrane, resulting in cytoplasmic acidification (Baker-Austin and Dopson, 2007). Although acetic acid concentrations were not monitored in the IC during incubation, it could be speculated that *Desulfosporosinus* oxidized glycerol incompletely to acetic acid, which could subsequently be used by *Acididesulfobacillus* as organic carbon source. This prevents toxic build-up of this organic acid, benefiting not only *Acididesulfobacillus* but also the other SRB.

The high relative abundance of reads classified as *Desulfosporosinus* in all IC from BR0 and BR21, including the cool-stored ones, suggested that SRB already were dominant community members in the original Brunita pit lake sediments. Previously, sequences classified as *Desulfosporosinus* were detected in the monimolimnion of BR (pH 4.5), albeit at lower relative read abundance (0.5%) than others belonging to the SRB-containing genera *Desulfomonile* (12.3%) and *Desulfurispora* (3.5%) (Sánchez-España et al., 2020a).

In contrast to the cool-stored IC from BR0 and BR21, no SRB taxa were detected at high relative abundance in the sequenced reads from the cool-stored TI IC. Rather, *Rhodanobacter* was the most abundant genus detected in the sequenced reads from this IC. Members of *Rhodanobacter* are capable of nitrate reduction (Green et al., 2012), suggesting that nitrate reduction could be a dominant metabolism at the original sampling site (Koh et al., 2015), and hence that sulfate reduction was not a significant microbial metabolic process at that sampling location. However, during the 18-months incubation of TI sediments, SRB became the most abundant taxa detected in the sequenced reads regardless of glycerol amendment, and pH profiles showed a similar development in both IC. Interestingly however, glycerol amendment appeared to strongly determine which SRB genus became most abundant — in the unamended IC *Desulfosporosinus* was the dominant genus, whereas it was nearly absent in the glycerol-amended IC and instead sequences affiliated to the novel species *Acididesulfobacillus*, were detected at high relative abundance. This difference was striking and could not be explained, for example, by a significant difference in pH between columns. It could be speculated that in TI, glycerol amendment provided a competitive advantage to *Acididesulfobacillus*, enabling it to outcompete *Desulfosporosinus*, while the organic carbon compounds present in the original TI sediments favored *Desulfosporosinus*.

In interpreting these results, it must be kept in mind that in order to avoid disturbance of the incubations, the microbial community was only determined after 18 months of incubation, masking any developments in the microbial community composition, especially in the early phases. This is especially relevant given the knowledge gap that exists regarding the microbial processes involved in the onset of pH neutralization and sulfidogenesis. As mentioned above, only moderately acidophilic or acidotolerant SRB have been isolated so far. In APLs, at pH 3–4 the activity of SRB has been reported to be rather low, while in the same range Fe(III) reduction is known to be 100-fold higher (Wendt-Potthoff et al., 2012). It could be therefore speculated that microbially induced neutralization is initiated by acidophilic Fe-reducing bacteria (FeRB) genera, when possible, also by nitrate reducing genera, after which SRB species (acidotolerant to neutrophilic) can establish due to improved physicochemical conditions.

The presence of reduced Fe(II) in the columns (evidenced by Fe sulfides and coherent with low ORP/Eh values of the solutions) and substantial increase of SO_4_^2−^ at the end of incubation might be an indirect evidence for the presence of FeRB and their active metabolism, which has a competitive advantage over SRB in oligotrophic systems at given pH (Diez-Ercilla et al., 2014). At the pH range at the beginning of incubation, between 2.4 and 4.5, the microbial reduction of Fe(III) (reaction 3; Bao et al., 2018) might be the dominant acidity-consuming process. This is in agreement with the higher abundance of FeRB (e.g., *Acidithrix* (Jones and Johnson, 2015), *Ferrithrix* (Johnson et al., 2009), and *Acidiphilium* (Johnson and Bridge, 2002)) in cool-stored IC, which might represent the least evolved microbial consortium compared to untreated and gly-amended ones.

$$4\text{Fe}{\left(\text{OH}\right)}_{3}+{\text{CH}}_{2}{\text{O}+8\text{H}}^{+}\to 4{\text{Fe}}^{2+}+{\text{CO}}_{2}+11{\text{H}}_{2}\text{O}$$ (3)

The presence and activity of nitrate-reducing microorganisms, suggested by the increase of dissolved ammonia at the water sediment interface of TI unamended column in the first 3 months (Figure 5C), might be another microbial metabolism with importance in acidity neutralization, following reaction 4 (Strohm et al., 2007), although the variation of dissolved NO_3_^−^ was rather low during the incubation. This could be related to the presence of *Rhodanobacter*, which were reported to grow below pH 4.0 and to be capable of complete denitrification (Green et al., 2012; Prakash et al., 2012). *Rhodanobacter* were abundant in the sequenced reads from the cool-stored TI IC. Although they were not abundant in the untreated and glycerol amended IC after 18 months, the increase in dissolved ammonium in the first 3 months could have due to *Rhodanobacter*, being afterwards outcompeted in numbers by SRB. It cannot be excluded, however, that they became abundant during incubation at 4°C, and this therefore remains speculative.

$${\text{C}}_{6}{\text{H}}_{12}{\text{O}}_{6}{+3\text{NO}}_{3}^{-}{+6\text{H}}^{+}\to {6\text{CO}}_{2}{+3\text{NH}}_{4}^{+}{+3\text{H}}_{2}\text{O}$$ (4)

### 4.2 Effects of SRB-mediated sulfidogenesis on acidity attenuation and metal concentration

The initial stage in all IC showed no evolution or very slow alkalinization, as well as slow reduction of the ORP (both ORP and pH showed an inverse relationship). Given the pH around 2.3 at FC and GU systems, a pH increase was only achieved when H^+^-consuming microbial metabolism, likely by FeRB such as *Acidithiobacillus* (Liao et al., 2009), outperformed the precipitation of early Fe(III) oxyhydroxysulfates, predominant in this pH range (Reaction 5; Sánchez-España, 2017), which released 22 mol of protons per each mole of precipitate:

$${8\text{Fe}}^{3+}{+\text{SO}}_{4}^{2-}{+14\text{H}}_{2}\text{O}\to {\text{Fe}}_{8}{\text{O}}_{8}\left({\text{SO}}_{4}\right){\left(\text{OH}\right)}_{6}{+22\text{H}}^{+}$$ (5)

Additionally, the same pH imposes serious limitations for bacterial metabolism. It was proposed that moderately acidophilic and acidotolerant SRB are able to establish and thrive in AMD sediments through the protection in micro-niches with less extreme conditions (Koschorreck, 2008), although this has long been a matter of debate. The finding of SRB in the water column of acidic pit lakes suggests that the microniche protection might not be essential for truly acidophilic SRB to thrive in these conditions (Sánchez-España et al., 2020a; van der Graaf et al., 2020).

The microbial activity was promoted at the water-sediment interface, in the first centimeters, as shown by the pH and ORP profiles (Figures 2, 3). The APLs from which the sediment and water samples were taken also showed an increase of pH and decrease of ORP with depth (Sánchez-España et al., 2020a). If this was due to a water-sediment interface increased activity or to an electron redox succession with depth, favoring sulfate reduction at deeper layers, need to be further investigated. Generally, fine silty and clayish sediment with up to 86% of particles >0.5 mm in BR0 (granulometric distribution for shore IC shown in Supplementary Figure S14), and even finer sediment in deeper BR21, enriched with nutrients via adsorption of P and N to clays and Fe minerals (Simpson et al., 2019) during decantation, make up a perfect environment with low effective porosity and rather isolated from the harsh characteristics found in the overlaying water column. This low effective porosity due to a very fine sediment in FC18 and BR21, in comparison to other ICs, might be responsible for the characteristic patchy-like black sediment growth. Additionally, the reduced size of each pocket should have facilitated the local alkalinization and therefore the onset of acid-tolerant microorganism´s growth subsequently expanding vertically. This evolution appears to have occurred in GU0 glycerol-amended IC, where the neutralization was restricted to the top centimeters of the sediment and then began expanding towards the water column.

In close vicinity to these dark seams, H_2_S odor could be detected. However, the quantitative measurements through dimethyl-p-phenylenediamine reaction (Hach LCK653) showed H_2_S concentration below the limit of detection, stated as 0.1 mg/L. The olfactory detection of H_2_S is reported to be in the order of 0.5 ppb (Hoshika et al., 1993), clearly above the detection limit of the cuvettes of 0.1 mg/L, suggesting that the experimental results might have been influenced by analytical interference with other dissolved species. However, the abundant Zn, Cu and/or Fe sulfides unequivocally allow inferring the presence of biogenic hydrogen sulfide in the medium. These mostly spherical aggregates appeared in close relation to either clean or mineralized microorganisms in the water column and sediment. Even though, it was mostly abundant within the first 2 cm of the sediment, commonly not coinciding with the pH neutralization peak.

In those ICs where the first schwertmannite buffer was surpassed, the recrystallization towards jarosite was the only abiotic H^+^-consuming process (Sánchez-España, 2017), while further aging to goethite (pH > 3; Miyata et al., 2018), the precipitation of sulfides (pH above 4.0) and formation of Al hydroxysulfates at pH > 4.0 (Sánchez-España, 2017) all imply acidity release. It must be noted though that the kinetics of these mineral transformations is often much slower compared to the microbial metabolism. This is illustrated indirectly by the P profile (Figure 5A): in the case of GU0, even in spite of microbial catalysis of Fe(III) reduction of schwertmannite by *Acidiphilium* and *Acidithiobacillus* (Bao et al., 2018), P release began only after about 3 months and reached a peak value after 12 months in untreated columns. Therefore, the net neutralization, observed in all ICs advocates for the great extent of proton consumption involved in bacterial Fe reduction (Reaction 3) and sulfate reduction (Reaction 2), rather than mineral formation.

The only exceptions to this general rule were the Brunita ICs, in which we detected the presence of carbonates in both shore and deep sediments, inducing fast chemical neutralization from the start of incubation, creating a more appropriate niche (pH 5.6 just the next day after the homogenization) for both acidotolerant and even neutrophilic microorganisms. This initial pH shift likely enabled a very intense microbial growth, which can be speculated to be predominantly SRB (smell and abundant sulfide formation), resulting in a pH 6.0–6.3 in BR21 and up to 7.8 in BR0 with much shorter lag phase in IC with glycerol amendment. Given the eutrophic state of the original Brunita pit lake we observed important neutralization in unamended IC, though slower and with less intensity and no evolution in cool-stored IC, clearly shown in the dissolved CO_2_ profile of BR0 (Figure 4C).

In the amended ICs from the different sites, the peak values of pH and ORP were achieved just after 14 days for BR21 comparing to 3 months for FC18 and 9 months for FC0 and GU0, after which we detected the beginning of an acidifying trend. In the IC from FC18, two vertically separated peaks in pH were detected. We hypothesize that the decrease might be related to the exhaustion of added organic carbon and a subsequent change of the balance between the acidity-consuming bacterial metabolisms and the acidity-releasing mineral precipitation. This could be in agreement with the evolution of TI IC, which contained large amount of algae, providing organic carbon throughout incubation. BR0 showed no setback either, which might be related to the high pH (>7.3), which could enable other neutrophilic microbial metabolisms to compensate the acidification from mineral precipitation.

This high microbial activity and the consecutive increase in pH had a clear impact on metal concentrations in these systems, since the reaction of biogenic H_2_S with dissolved metal(oid)s (e.g., Cu, Zn, Cd, As) led to a very important removal of these metals from the aqueous solutions (Figure 6, Supplementary Figures S8–S13). In addition, an important issue needs to be considered as regards to the main toxic elements, As and Cd. EDX analysis of Fe oxyhydroxysulfates showed incorporation of some of these trace elements (*data not shown*). It is widely reported that during the dissolution and recrystallization of schwertmannite, this mineral releases ionic species initially adsorbed onto the surface or in its’ structure (Jones et al., 2006; Schoepfer and Burton, 2021; Zhang et al., 2022). On the one hand, the release of P (Figure 5A) to the interstitial space might be beneficial for the microbial growth in oligotrophic systems, but the release of As and Cd, commonly adsorbed onto schwertmannite and jarosite (Burton et al., 2009; Burton et al., 2021), would be hazardous to the local ecosystem. From the biotechnological point of view, it is therefore crucial to balance the re-solubilization processes with SRB-mediated sulfide precipitation. The formation of metal sulfides is also beneficial for some trace elements; for example, ZnS was proved to incorporate Cd (*not shown*), so that the concentration of this metal showed a generalized decreasing trend over time (Figure 6, Supplementary Figure S12), as opposed to the trend observed for As.

## 5 Conclusion

This study shows that biosulfidogenesis in oligotrophic AMD environments can be triggered by glycerol amendment, stimulating indigenous SRB, further supporting its potential as a relatively low-cost remediation solution for AMD. It is demonstrated that ICs built with oligotrophic sediment and water showed little evolution without glycerol amendment, while biosulfidogenesis occurred at very significant rates in eutrophic BR21 and BR0, eutrophicated TI, as well as in the glycerol-amended IC built from oligotrophic sites. This was inferred from the abundance of known SRB taxa detected through 16S rRNA gene amplicon sequencing at the end of incubation, the dissolved CO_2_ evolution, the removal of different metals (e.g., Cu, Zn, As, Cd) from the aqueous phase, and the formation of biogenic Zn, Cu and Fe sulfides. This study supports that biosulfidogenesis in the natural environment is highly constrained by the availability of suitable carbon sources for the indigenous SRB consortia. The results support the tight link between biosulfidogenesis by SRB and metal attenuation in AMD-affected areas. The highest activity appeared restricted to the first and most reactive centimeters of the sediments, and biosulfidogenesis expanded upwards into the overlaying water column, as evidenced by the ICs. The presence of carbonates in sediments from BR0 and BR21 has enhanced biosulfidogenesis through pH increase after their dissolution, allowing the establishment of communities of moderately acidophilic to acidotolerant bacterial groups (e.g., *Desulfurispora*, *Desulfovibrio*, *Desulfosporosinus* and *Acididesulfobacillus*), further enhancing the biosulfidogenesis, as SRB known to data are moderately acidophilic or acidotolerant.

## Data Availability

The datasets presented in this study can be found in online repositories. The names of the repository/repositories and accession number(s) can be found below: https://www.ebi.ac.uk/ena, PRJEB52361 (secondary: ERA12717712).
