# Supplementary material for: Glycerol amendment enhances biosulfidogenesis in acid mine drainage-affected areas: An incubation column experiment
Source: Front Bioeng Biotechnol. 2022 Aug 29;10:978728. doi: 10.3389/fbioe.2022.978728 (PMC9464833; doi:10.3389/fbioe.2022.978728)
Supplement: Supplementary file 1 [file DataSheet1.docx]

Supplementary Material

## Supplementary Figures


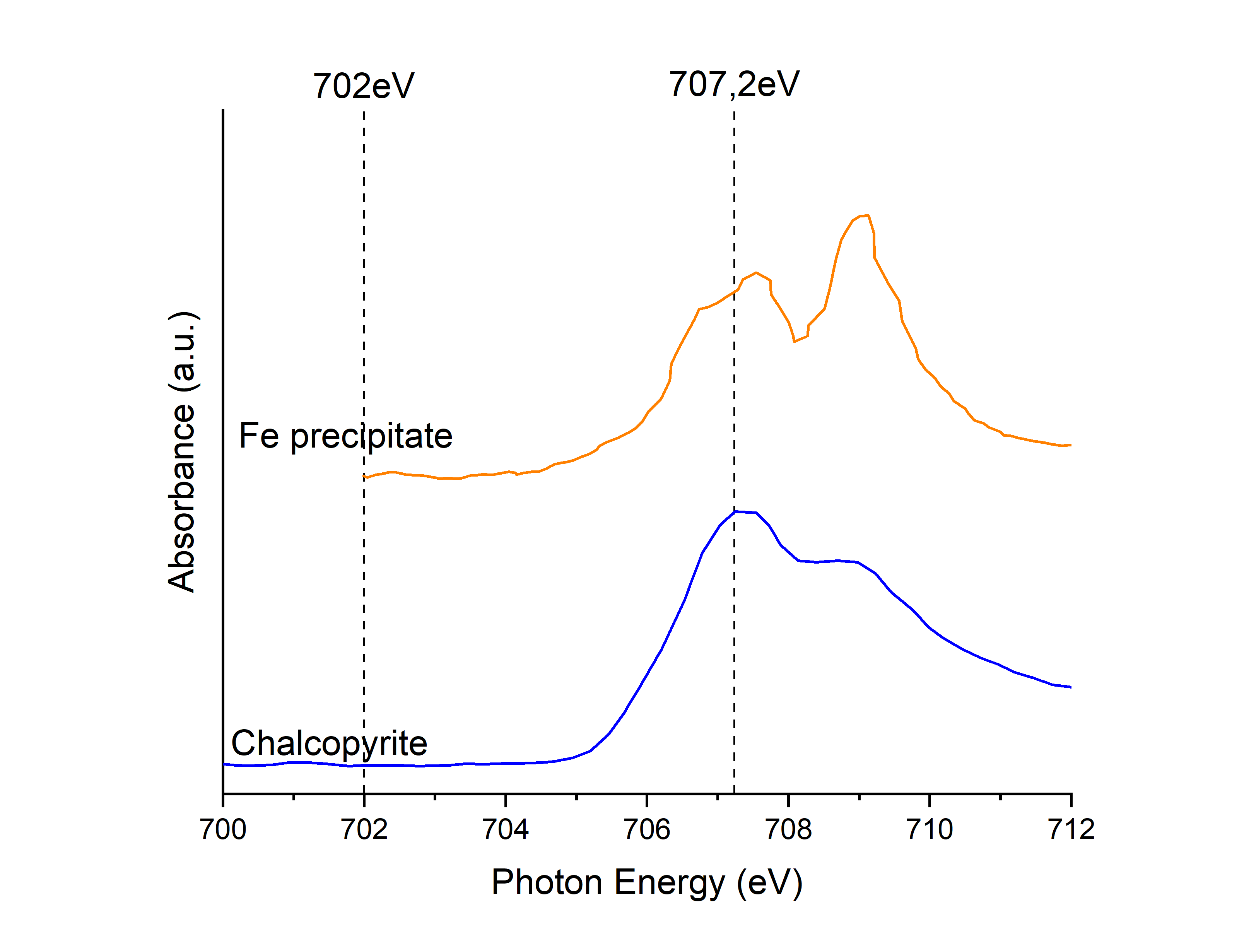


**Supplementary Figure 1.** Integrated Fe L_3_-edge of a reference sample (pure chalcopyrite) and Fe precipitate. Dotted line shows the pre-edge and edge energy position evidencing the big difference in absorbance and therefore in contrast.


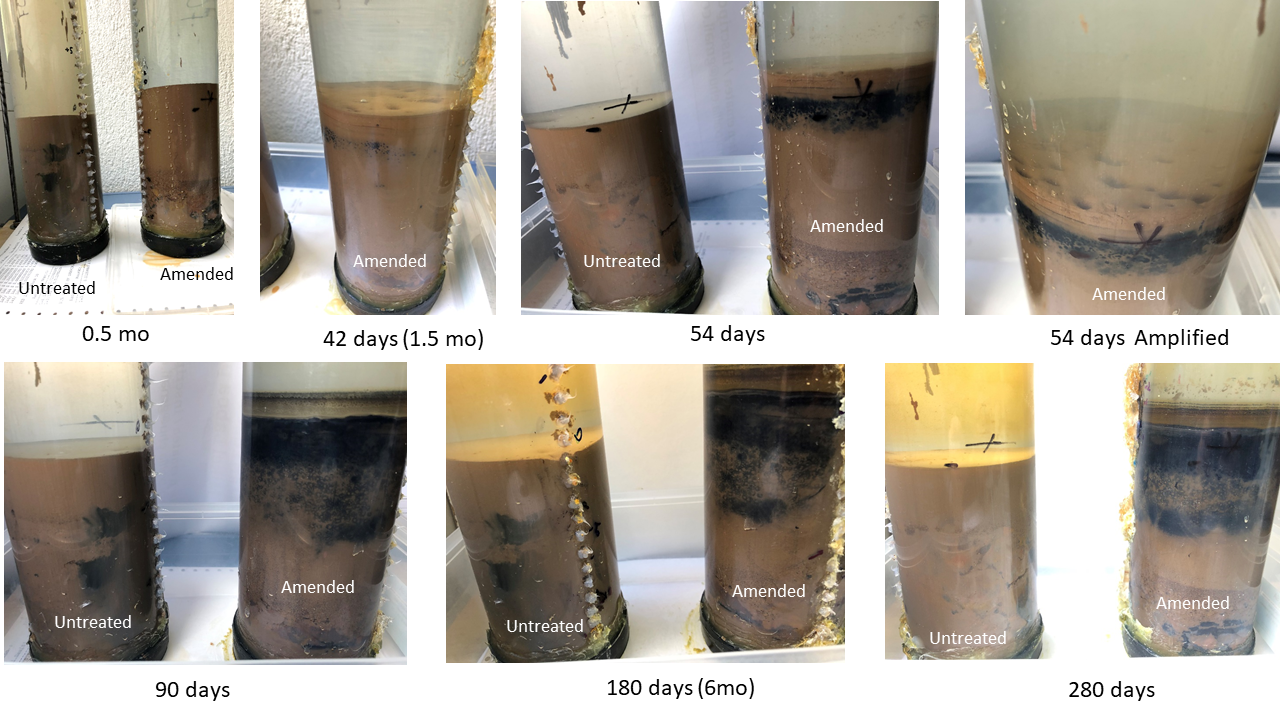


**Supplementary Figure 2.** Visual changes in different incubation columns built with core sediment from Filón Centro 18m (monimolimnion).


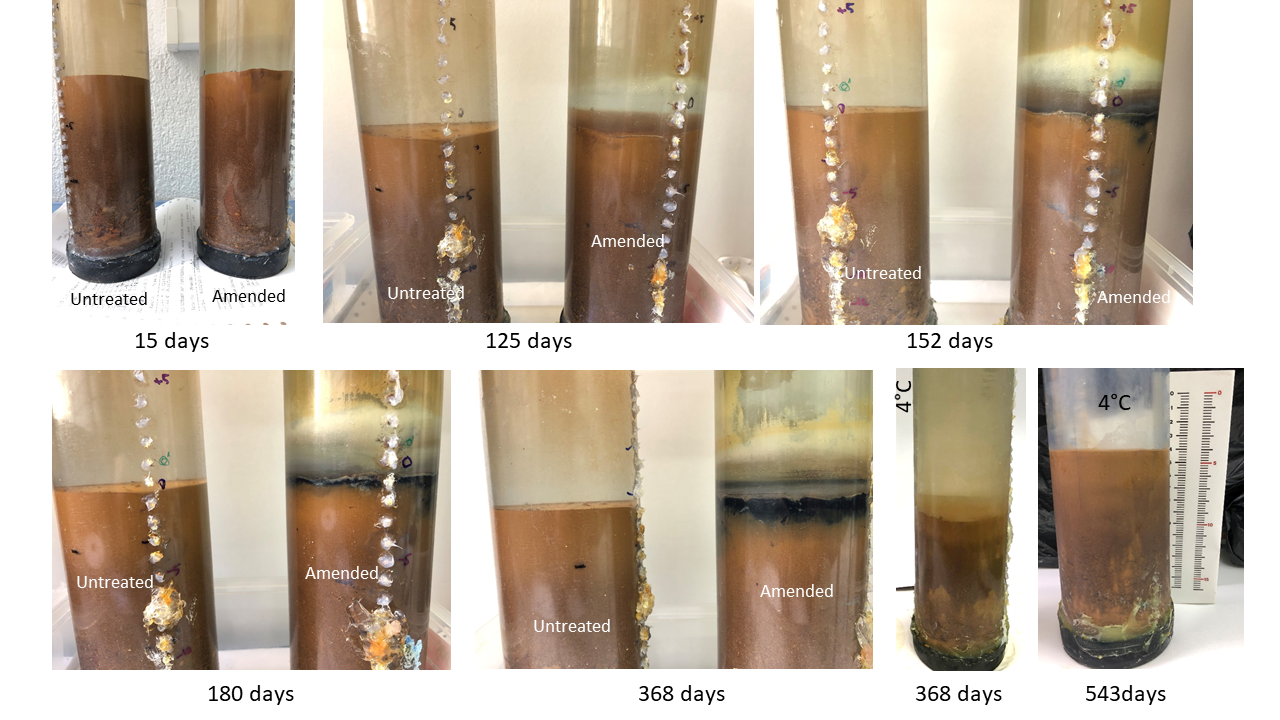


**Supplementary Figure 3.** Visual changes in different incubation columns from Filon Centro 0m (mixolimnion).


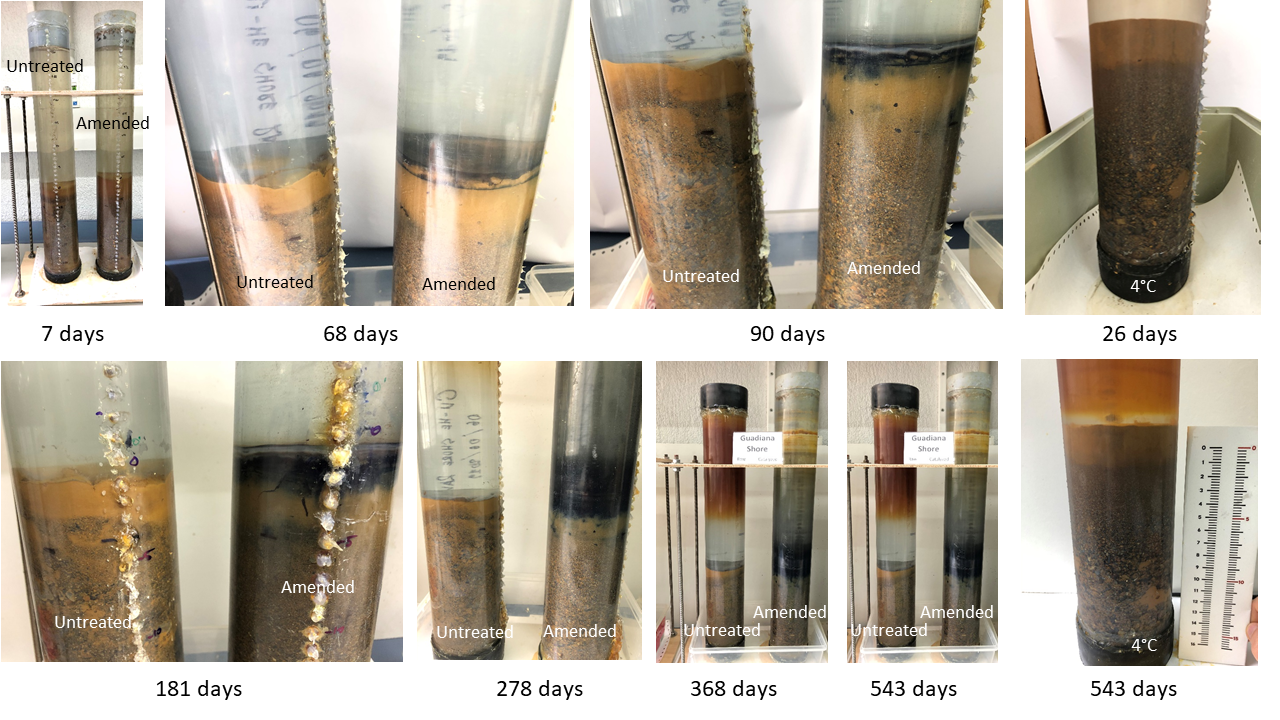


**Supplementary Figure 4.** Visual changes in different incubation columns from GU 0m (mixolimnion).


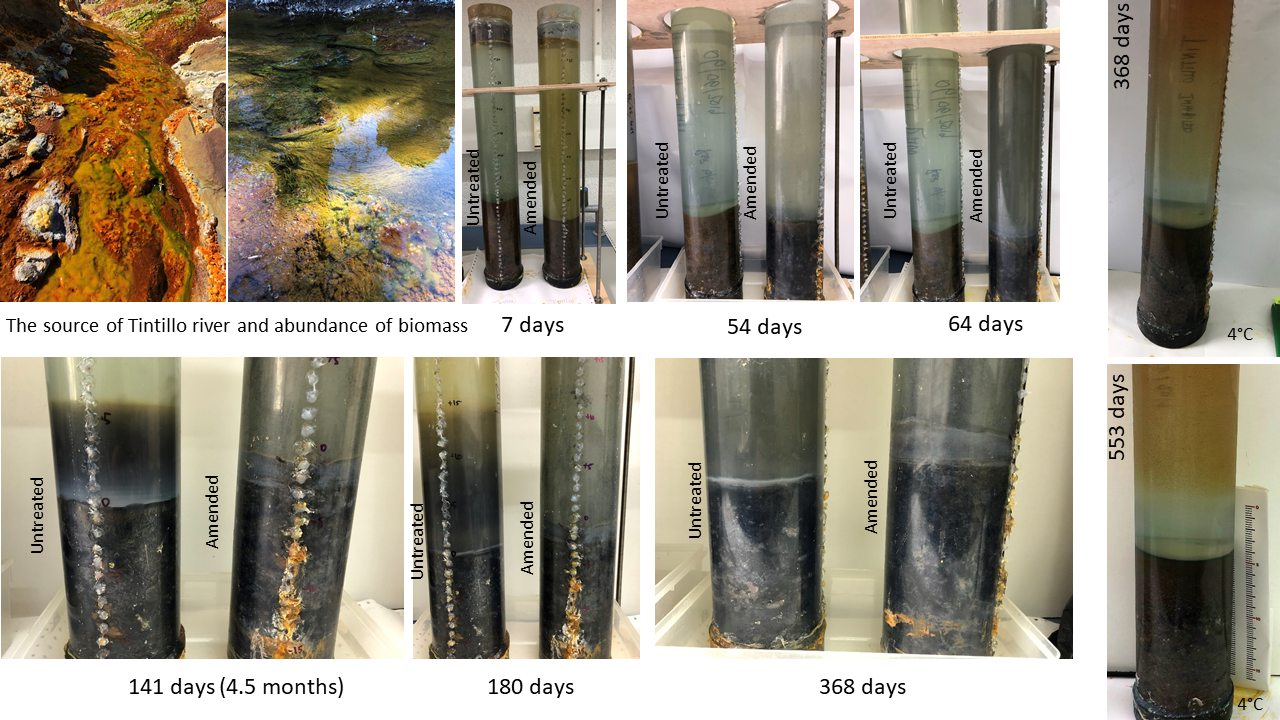


**Supplementary Figure 5.** The source sediment from Tintillo acidic stream with abundant biomass and visual changes in different incubation columns (mixolimnion/stream).


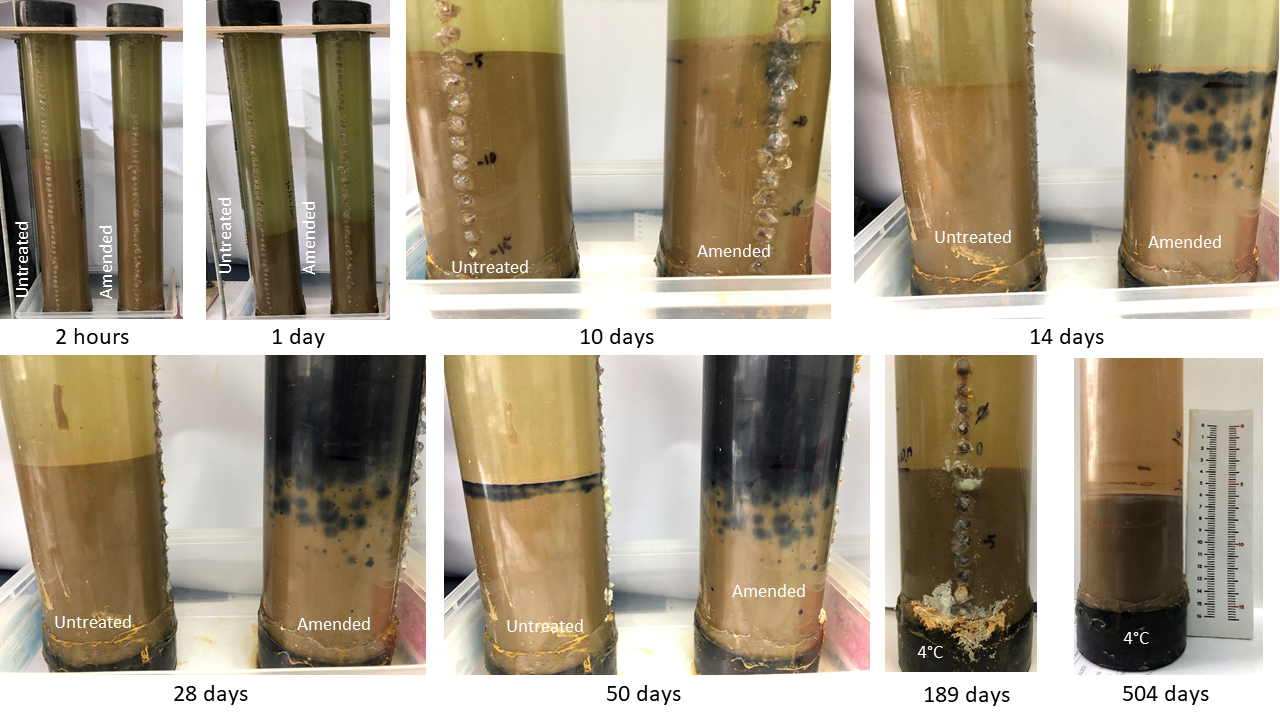


**Supplementary Figure 6.** Visual changes in different incubation columns built with core sediment from Brunita 21m (monimolimnion).


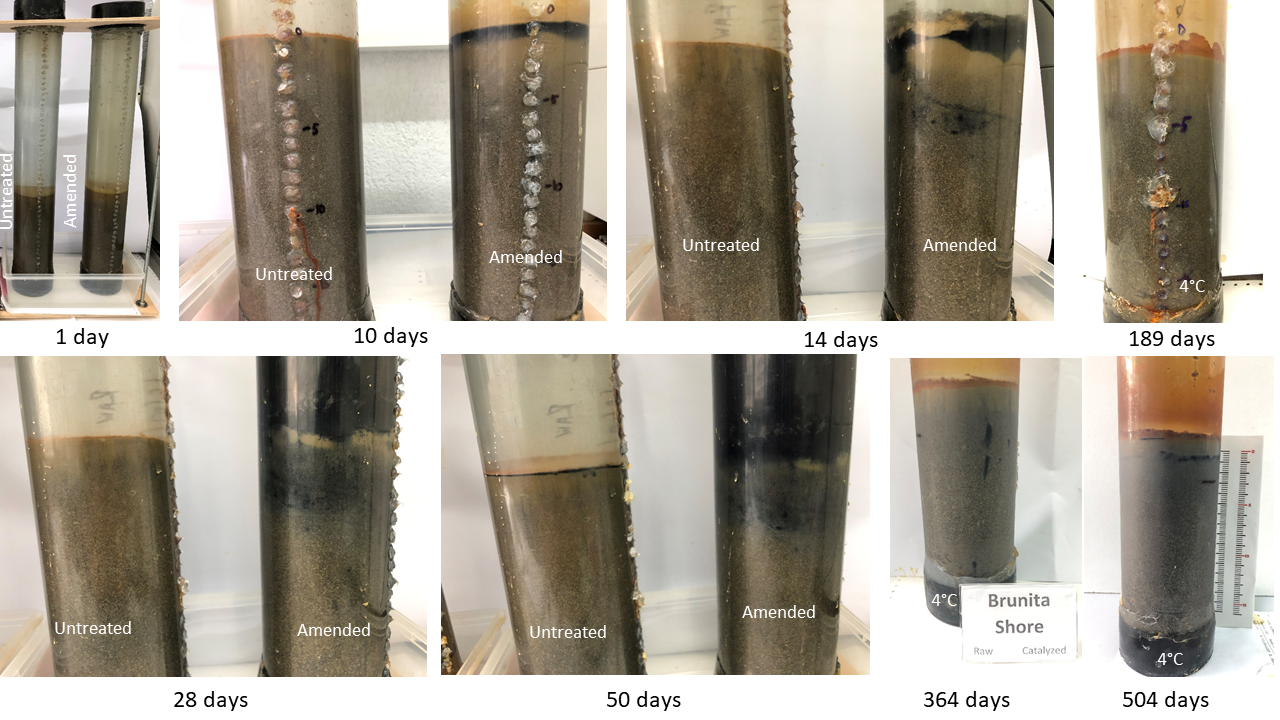


**Supplementary Figure 7.** Visual changes in different incubation columns built with core sediment from Brunita 0m (mixolimnion).

**
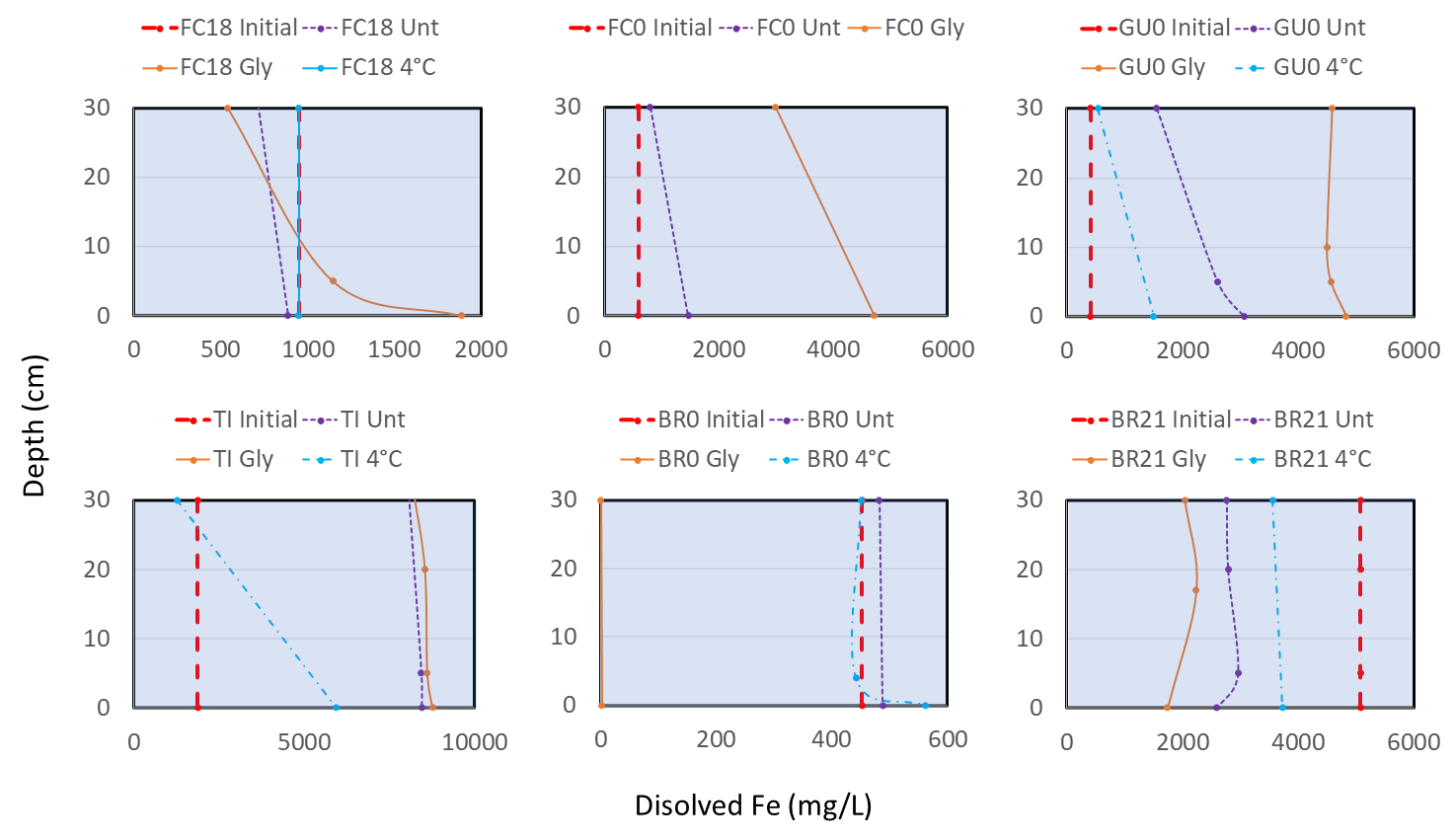
**

**Supplementary Figure 8.** *Iron concentration profiles obtained at different points of the water column overlaying the sediment. The concentration at the beginning of incubation (red dashed line) is compared to the concentration of these elements after 18mo of incubation with different treatment.*

*
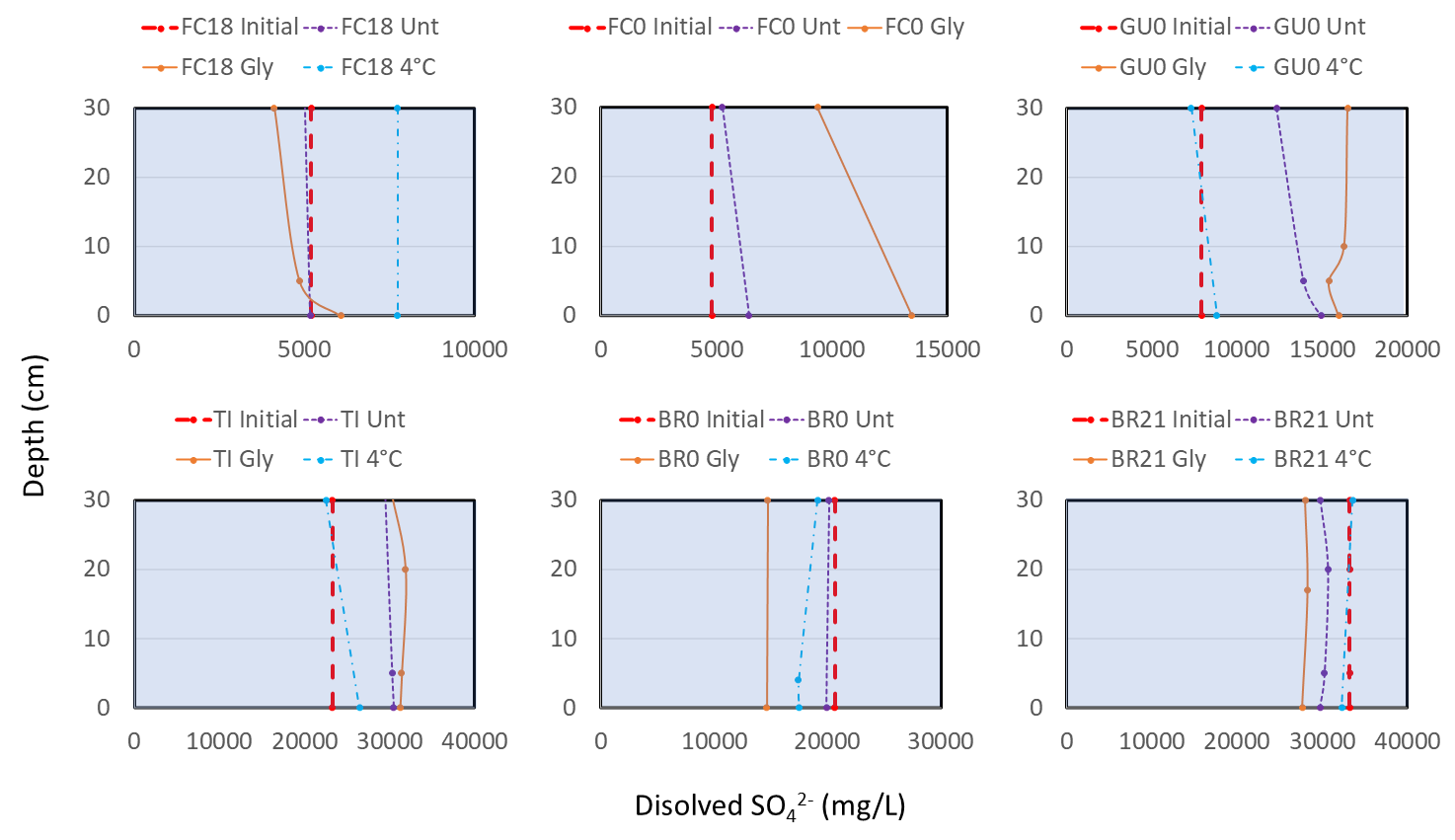
*

**Supplementary Figure 9.** *Sulfate concentration profiles obtained at different points of the water column overlaying the sediment. The concentration at the beginning of incubation (red dashed line) is compared to the concentration of these elements after 18mo of incubation with different treatment.*


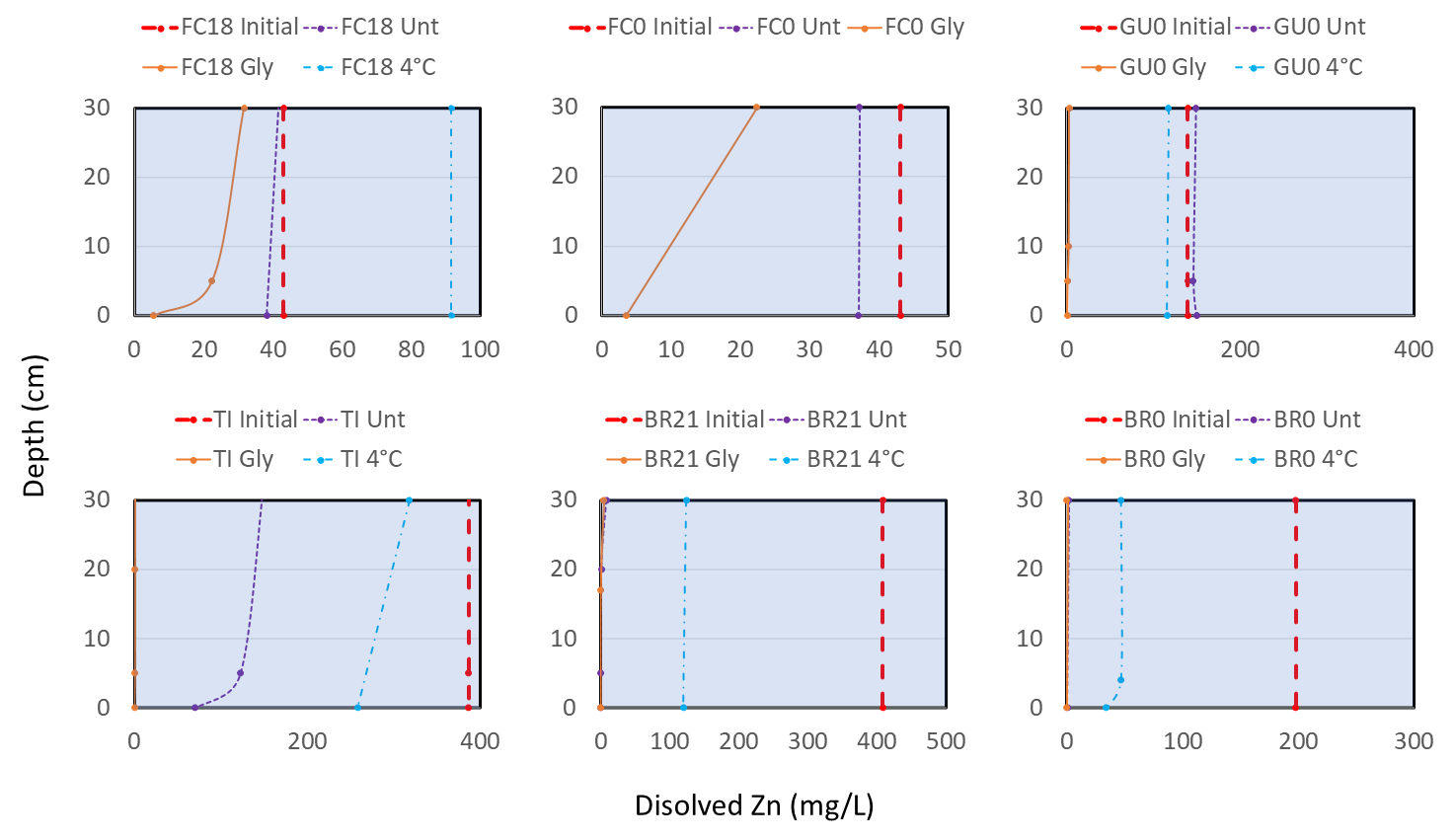


**Supplementary Figure 10.** *Zinc concentration profiles obtained at different points of the water column overlaying the sediment. The concentration at the beginning of incubation (red dashed line) is compared to the concentration of these elements after 18mo of incubation with different treatment.*


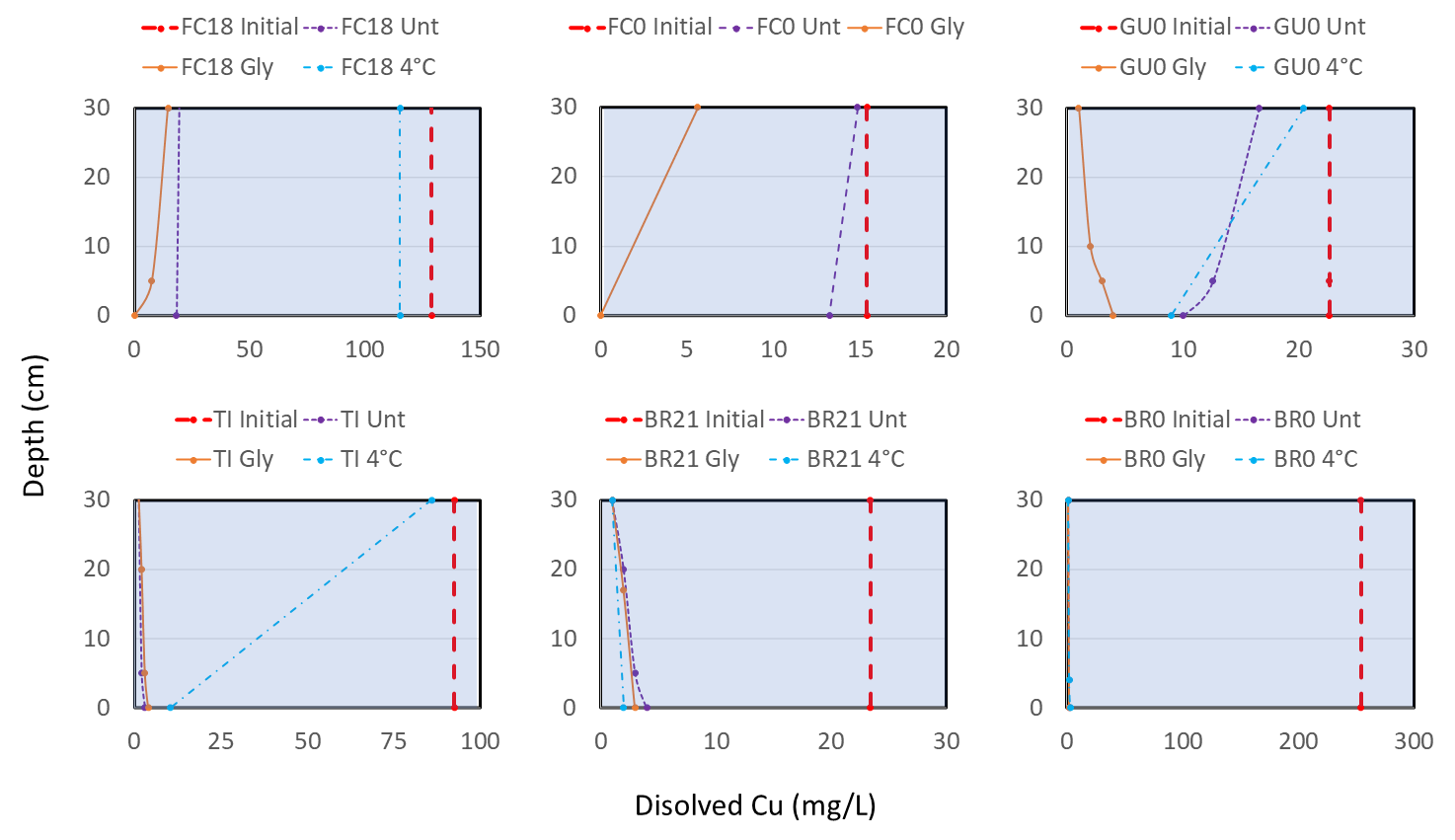


**Supplementary Figure 11.** *Copper concentration profiles obtained at different points of the water column overlaying the sediment. The concentration at the beginning of incubation (red dashed line) is compared to the concentration of these elements after 18mo of incubation with different treatment.*


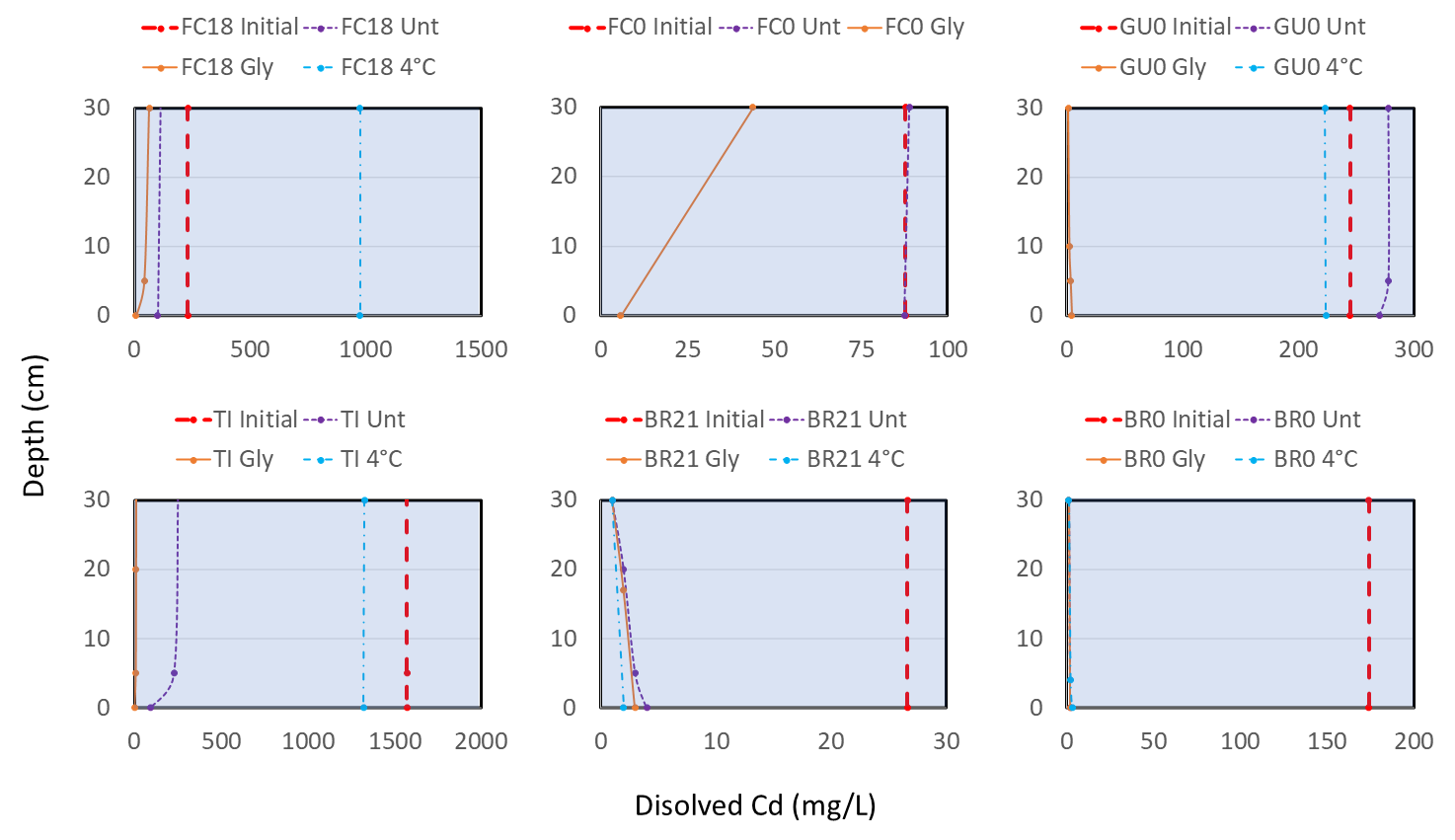


**Supplementary Figure 12.** *Cadmium concentration profiles obtained at different points of the water column overlaying the sediment. The concentration at the beginning of incubation (red dashed line) is compared to the concentration of these elements after 18mo of incubation with different treatment.*

**
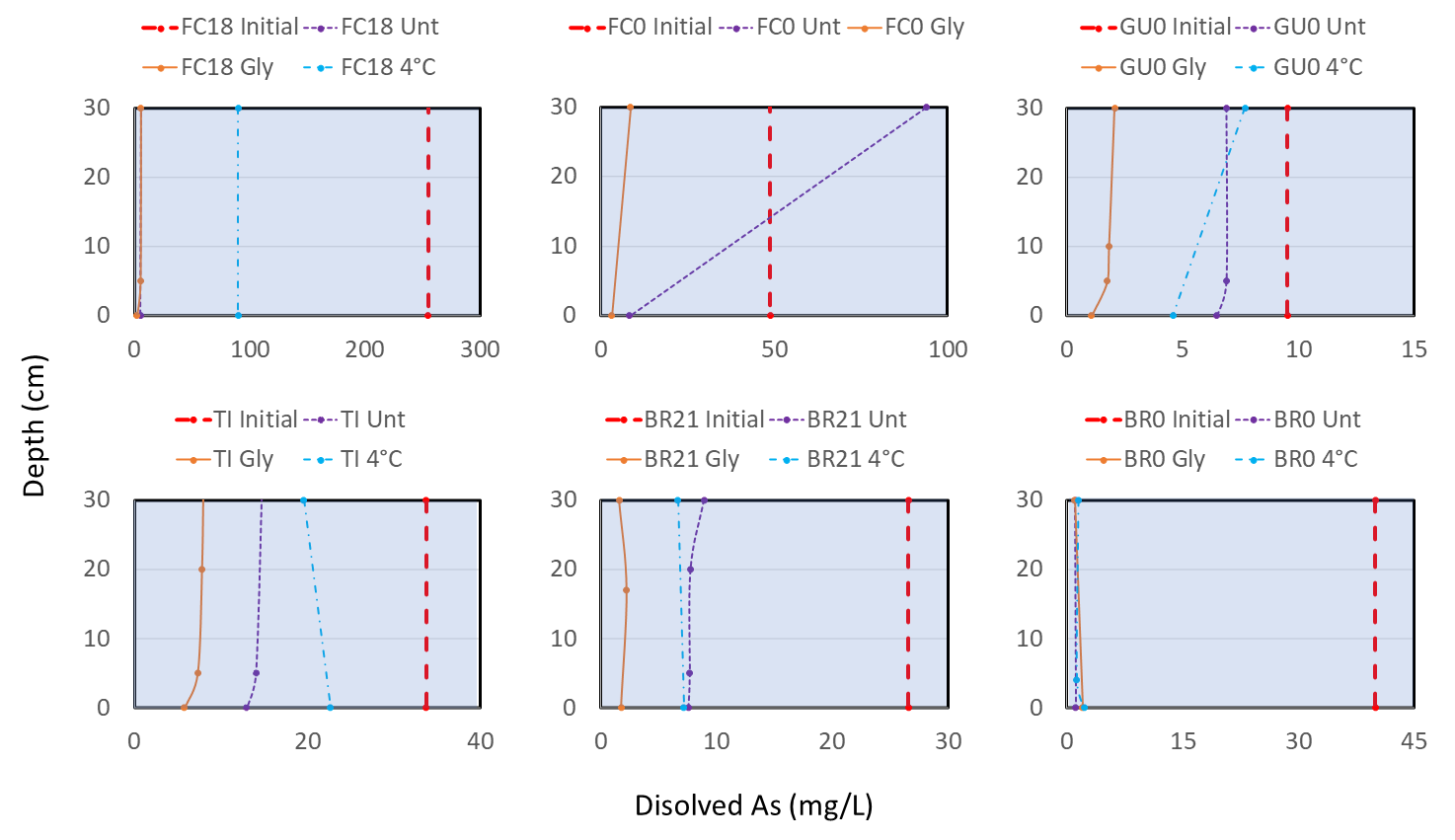
Supplementary Figure 13.** *Arsenic concentration profiles obtained at different points of the water column overlaying the sediment. The concentration at the beginning of incubation (red dashed line) is compared to the concentration of these elements after 18mo of incubation with different treatment.*

**Supplementary Figure 14.** Granulometric distribution of initial sediment used for incubation columns.
